# Applying a risk assessment guided evaluation for verifying comprehensive two-dimensional gas chromatography to analyse complex pharmaceuticals

**DOI:** 10.1007/s00216-023-05093-9

**Published:** 2023-12-21

**Authors:** Lukas Schwalb, Ole Tiemann, Uwe Käfer, Christopher Paul Rüger, Thomas Gröger, Ralf Zimmermann

**Affiliations:** 1 Joint Mass Spectrometry Centre (JMSC), Chair of Analytical Chemistry, University of Rostock, Rostock, Germany; 2 Joint Mass Spectrometry Centre (JMSC), Cooperation Group “Comprehensive Molecular Analytics” (CMA), Helmholtz Zentrum München GmbH, German Research Center for Environmental Health, Neuherberg, Germany; 3 Department Life, Light & Matter (LLM), University of Rostock, Rostock, Germany; 4 Present Address: Leibniz-Institute of Tropospheric Research (TROPOS), Leipzig, Germany

**Keywords:** GC × GC, HR-MS, Risk assessment, Non-biological complex drugs, Sodium bituminosulfonate, FT-ICR MS

## Abstract

The reliability of analytical results is critical and indispensable when applied in regulated environments such as the pharmaceutical industry. Therefore, analytical workflows must be validated. However, validation guidelines are often designed for quantitative targeted analysis and rarely apply to qualitative untargeted approaches. In this study, we employ a risk assessment approach to identify critical parameters which might influence the qualitative results derived by online derivatisation — comprehensive two-dimensional gas chromatography coupled to a high-resolution time-of-flight mass spectrometer (GC × GC-HR-ToF–MS) for the analysis of the active pharmaceutical ingredient (API) sodium bituminosulfonate (SBS). To show the complexity and feasibility of such an approach, we focus on investigating three potential risk factors: sample preparation, vapourability, and the thermal stability of sulfonates. Through the individual evaluation of these potential risk factors due to the application of sample preparation approaches and thermal gravimetric analysis (TGA), we demonstrate the high derivatisation efficiency and repeatability of the online derivatisation method and confirm the absence of derivatisation-induced side reactions. In addition, we also show the potential thermal instability of an incompletely derivatised API. To address the limitation of these individual assessments, we applied a holistic evaluation step with negative electrospray ionisation Fourier transform ion cyclotron resonance mass spectrometry (ESI( −) FT-ICR MS) as an orthogonal technique. This confirms that most of the API is detected via the presented GC-based method. Thereby, we demonstrated the practical feasibility of the risk assessment-based approach to ensure the validity of the qualitative data for a complex untargeted method.

**Graphical Abstract:**

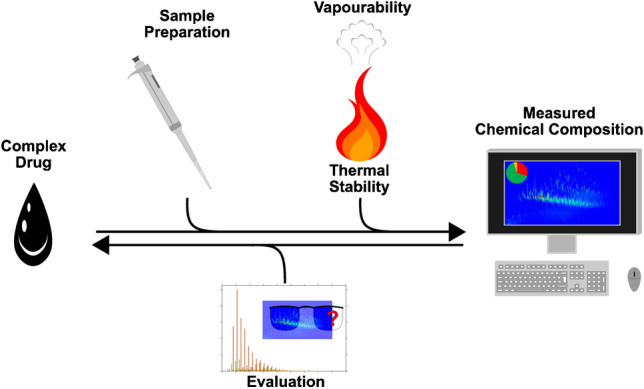

**Supplementary Information:**

The online version contains supplementary material available at 10.1007/s00216-023-05093-9.

## Introduction

A critical aspect in the description of pharmaceuticals is the determination of their exact chemical composition, which is usually investigated by several physicochemical methods during drug development and must be reported and included in the specification [1]. The composition determines the physicochemical properties and the pharmacological effects of a drug. Therefore, regulatory authorities are also beginning to focus on the chemical composition of complex biological and non-biological drugs [2, 3], for which the exact composition has not yet been clearly identified, and it has been assumed and defended so far that they are not resolvable by physiochemical means [4].

The qualitative and quantitative identity of the active pharmaceutical ingredient (API) is the basis of a quality control (QC) method, which is then applied to assure the quality of different batches during the production process. These methods are targeted and determine the presence, absence, and/or concentration of APIs, critical impurities, and specific marker compounds. The requirements for implementing a QC method in the pharmaceutical environment are high, and multiple aspects must be addressed and evaluated. This evaluation is performed and documented in the validation phase. The validation parameters are described by the pharmacopoeia or guideline Q2 of the International Council for Harmonization (ICH). Their level of detail and extent differ depending on the applied method [5]. However, this guideline only covers targeted analysis and does not address qualitative untargeted approaches, which might be better suited or even required to study very complex matrices.

In contrast to targeted analysis, where a specific analyte or set of analytes are defined, untargeted approaches aim to get an overview of the qualitative composition of a complex sample without discriminating key compounds or even groups of compounds. Within this untargeted analysis, characteristic compounds and patterns can be addressed. These methods enable the identification of meaningful and reliable targets for QC methods or provide a chemical fingerprint for the distinction of competitors or copycat products [6, 7].

One method particularly suitable for untargeted analysis of volatile and semi-volatile compounds is comprehensive two-dimensional gas chromatography (GC × GC). The technique achieves high separation performance with minimal demand on sample purification at the same time for very complex samples. It allows separation and access to thousands of compounds [8]. GC × GC became an essential technique for the in-depth chemical description of complex matrices in various application fields [7, 9–12]. Often, GC × GC is applied as a screening and fingerprinting tool in addition to targeted techniques, and very recently, the first attempts have been made to standardise GC × GC analysis [13]. However, for the analysis of pharmaceuticals, very high standards must be fulfilled [1].

When using complex comprehensive and non-directional approaches, the measurement result must be considered to represent the sum of the matrix, sample treatment, and characteristics of the applied analytical system (e.g. physical limitations). In the case of untargeted methods, for which neither reference methods nor standard materials exist, it is also often difficult to distinguish between handling or analysing artefacts and sample information. Therefore, it is essential to identify possible interference and limitations before, during, and after the analytical method to study them and evaluate their impact on the results [14].

Although ICH guidelines are designed for targeted analysis, some procedures are universally applicable and can be used to evaluate untargeted analytical methods. A new guideline in the draft version will recommend a risk assessment for developing or assessing an analytical method in the pharmaceutical industry [14]. Here, potential influencing parameters are identified and considered for their impact [15]. Because of the theoretical nature of this approach, the determined main influencing parameters should be investigated empirically. These steps should be performed before the validation, in the method development, or after problems occur (e.g. results out of specifications). The evaluation of the critical parameter indicates the method’s specificity, precision, and robustness [14]. An alternative or additional evaluation method is the comparison to another analytical method. This approach is used for cross-validation in studies with multiple analytical sites in the regulated environment. This validation ensures the comparability of at least two analyses at diverse locations within the analytical workflow. Although the guideline requires at least one validated method, comparing two methods can improve the confidence of the results. For this purpose, ideally, the alternative analysis should be based on other physicochemical principles. This enables an evaluation of possible discrimination effects with an orthogonal analytical technique [5].

In a previous study, we analysed sodium bituminosulfonate (SBS), an API for various pharmaceutical products that are today tentatively allocable to non-biological complex drugs (NBCDs) [16–18]. Its origin as the sodium salt of a sulfonated shale oil distillate presented a high chemical complexity and challenges for a comprehensive analysis. We used online derivatisation to enable the applicability of GC × GC coupled to a high-resolution time-of-flight mass spectrometer (HR-ToF–MS) for the polar matrix. We compared the results with the analysis of the precursor to estimate the plausibility by considering the production process [16]. In this study, we propose a risk assessment to identify critical factors of the entire analytical procedure to verify the results considering the pharmaceutical background of the API. We focus on the crucial sample preparation steps and physical limitations of the applied chromatographic–mass spectrometric system to investigate their effects on the qualitative data. For this purpose, we compare different extraction and derivatisation methods and study the thermal behaviour of the different states of sulfonates (sodium salt, acid, and methyl ester) via thermogravimetric analysis, measuring the evolved gases via mass spectrometry (TGA-MS). Moreover, we cross-verify the results with the orthogonal technique of direct infusion negative mode electrospray ionisation Fourier transform ion cyclotron resonance mass spectrometry (DI ESI( −) FT-ICR MS). The study reveals significant influencing factors throughout the analytical procedure, investigates three critical parameters with complementary analytical methods, and performs a cross-verification of the complete method with an orthogonal technique. This allows us to verify the qualitative results and minimise the possibility that important compounds have not been detected. The verification is required to use the data in the pharmaceutical industry, for example, to design a QC method.

## Material and methods

### Sample material

Authentic production batches of sodium bituminosulfonate (Ichthyol-Natrium Hell®) were provided by Ichthyol-Gesellschaft Cordes, Hermanni & Co. (GmbH & Co.) KG, Germany. The derivatisation agent tetramethylammonium hydroxide (TMAH) (25% solution in water, Sigma-Aldrich, USA) and trimethylsilyldiazomethane (TMSDAM) (2 M in diethyl ether, Acros, USA) were used for the methylation of sodium bituminosulfonate. For the extraction and dissolving of the samples, toluene (> 99.5%, Roth, Karlsruhe, Germany), methanol (> 99.9%, Roth, Karlsruhe, Germany), and acetone (99.9%, Roth, Karlsruhe, Germany) were used. The adjustments of the pH values were performed with trimethylamine (> 99%, Sigma-Aldrich, France) and concentrated hydrogen chloride (37%, Roth, Karlsruhe, Germany). The used water was produced by a Milli-Q® water purification system (Merck, Darmstadt, Germany). Sigma-Aldrich made the standards used for the TGA: benzenesulfonic acid (98%, Sigma-Aldrich, USA), sodium benzenesulfonate (97%, Sigma-Aldrich, Switzerland), and methyl benzenesulfonate (98%, Sigma-Aldrich, India).

### Sample preparation

For the evaluation of the online derivatisation method, SBS and TMAH were mixed (1:2 V/V), diluted 1:10 with a mixture of water/methanol (1:1 V/V), and injected (0.5 μL); three different solid-phase and one liquid/liquid extraction were performed. The API was diluted 1:10 (V/V) with water and acidified with concentrated hydrogen chloride (HCl) to pH 1. The solid-phase extractions were done with a mixed-mode weak anion exchanger (WAX) (Phenomenex Strata-X-AW 33u polymeric weak anion), mixed-mode strong anion exchanger (SAX) (Phenomenex Strata-X-A 33u polymeric strong anion), and hydrophilic–lipophilic balance (HLB) cartridge (Waters Oasis® HLB). The cartridges were conditioned with 1 mL methanol, equilibrated with 1 mL acidified deionised water (pH 1), loaded with 0.5 mL of the acidified sample solution, and washed with 1 mL of acidified deionised water (pH 1). The elution was executed with 1 mL methanol/acetone (3:2, V/V), including 1 vol% triethylamine.

The liquid/liquid extraction was performed by adding 0.5 mL of the sample solution to 1 mL of concentrated HCl and 1 mL of toluene. The mixture was hand-shaken for about 10 s. After that, the mixture was placed and waited till the water, and toluene layers were separated. The toluene layer was obtained and dried under a gentle nitrogen stream. The dried extract was dissolved in 1 mL of a mixture of methanol/acetone (3:2, V/V), including 1 vol% of trimethylamine.

The extracts were derivatised by adding 40 μL of TMSDAM to 100 μL of each extract. The solution was kept at room temperature for 1 h, subsequently stored at 4–8 °C, and measured within 7 days by injecting 1 μL of the solution.

### Comprehensive two‑dimensional gas chromatography

The GC × GC measurements were carried out on a Pegasus® GC-HRT 4D (LECO, St. Joseph, USA) with 60 m BP1 (0.25 mm internal diameter, 0.25 μm film thickness, SGE) column in the first dimension and 1.5 m BPX50 (0.1 mm internal diameter, 0.1 μm film thickness, SGE) in the second dimension. GC × GC parameters are similar to our previous publication [16] on the analysis of SBS. Detailed parameters are listed in Table 1.

**Table 1:** Applied GC × GC parameters for the measurement of the derivatised extracts of sodium bituminosulfonate

	GC × GC parameters
Injection temp (°C)	350
Constant flow (ml/min)	1
Split	1:10
Liner	Split liner (low pressure drop, glass wool)
Oven program:	
Start temp Heat rate 1 Heat rate 2 End temp	40 °C (0 min) 10 °C/min up to 120 (0 min) 1 °C/min 270 °C (0 min)
2nd oven offset (°C)	30
Modulator offset (°C) (relative to the 2nd oven)	15
2nd dimension time (s)	4.5
Total run time [min]	158
Solvent delay (s)	1600

The mass spectrometric method and the data handling are also similar to this publication [16]. In summary, ionisation by electron ionisation with 70 eV at 300 °C, ions are acquired in “high-resolution mode” between *m/z* 15 and 500 with an acquisition rate of 100 Hz. Perfluorotributylamine (PFTBA) was added continuously for internal mass calibration. A mass resolution of at least ≥ 25 k for *m/z* 218.9856 and a mass error below 1 ppm were achieved in all measurements. Acquired mass spectra were calibrated and processed with the ChromaTOF HRT software (v5.10, LECO, St. Joseph, USA), and a home-built MATLAB (R2020b, MathWorks Inc., MA, USA) script was used for further data visualisation.

### Thermogravimetric analysis

The TGA-MS measurements were performed at a STAF449 F3 Jupiter thermo-balance (Netzsch, Selb, Germany) coupled with the same mass spectrometer used for the GC × GC analysis to measure the evolved gases. A deactivated, fused silica capillary (2.5 m length, 0.1 mm internal diameter, Agilent Technologies) and heated (250 °C) interfaces on both sides of the capillary were used for the coupling [18]. The TGA measurements were executed with a sample amount between 15 and 25 mg and measured in a ceramic crucible equipped with a loose lid (pinhole 50 μm diameter). The sample was heated from 40 to 1000 °C with a heat rate of 10 °C/min. The mass spectra were collected with an acquisition rate of 1 Hz in a mass window of *m/z* 40–500. The lower limit of the mass window was adjusted to detect the trace gases CO2 and SO2 but avoid the detection of the nitrogen atmosphere and unspecific fragments with low *m/z* values. While PFTBA is measured with the same mass resolution and mass error as reported for the GC × GC analysis in blank measurements, coupled to the TGA, we observed mass errors in average of 3.2 ppm for the samples. The measurements were further processed by a home-build MATLAB script (R2020b, MathWorks Inc., MA, USA). Sum formula and exact mass assignment were carried out with the following restrictions, #^12^C 0–30, #^1^H 0–62, #^14^N 0–0, #^16^O 0–6, and #^32^S 0–4; hydrogen was restricted between zero and the aliphatic limit C_c_H_2c+2_; a mass error of ± 5 mDa was allowed. If multiple elemental compositions were possible, the one with the lowest mass error was taken.

### Fourier transform ion cyclotron resonance mass spectrometry

The samples were analysed with a 7 T FT-ICR-MS equipped with an infinity cell (solariX, Bruker Daltonics, Bremen, Germany) and a minimum achieved resolving power of 400.000 at *m/z* 400 resulting from a 2 s transient. For the analysis, SBS was dissolved in water 1:10 V/V, and then 10 µL of each aqueous solution was further diluted with 990 µL of methanol to a final dilution of 2.5 ppm (V(sample)/V(solvent)). This solution was injected into an electrospray ionisation (ESI) ion source at 200 µL/h and a capillary voltage of 3.5 kV. Ionisation was operated in negative mode with a dry gas flow rate of 5.0 L/min at 200 °C and a nebuliser gas pressure of 1.5 bar. The instrument was calibrated by measuring arginine (0.02 mg/L in methanol) every workday. The calibration of the measurements was carried out in Data Analysis 5.1 (Bruker Daltonics, Bremen, Germany) using homologous rows that were previously manually identified. The mass lists were then extracted with a signal-to-noise ratio of 12. Further data treatment of the complex samples was performed in CERES, a self-written program based on MATLAB scripting (MATLAB R2020b). Mass spectra were blank corrected with an external background list containing interferences such as source background and impurities. Sum formula assignment was carried out with the following restrictions: #^12^C 4–100, #^1^H 4–200, #^14^N 0–0, #^16^O 0–6, #^32^S 0–4, double bound equivalent (DBE) 0–15, and mass error ± 1 ppm.

## Results and discussion

### Chemical characterisation of sodium bituminosulfonate

**Fig. 1:**
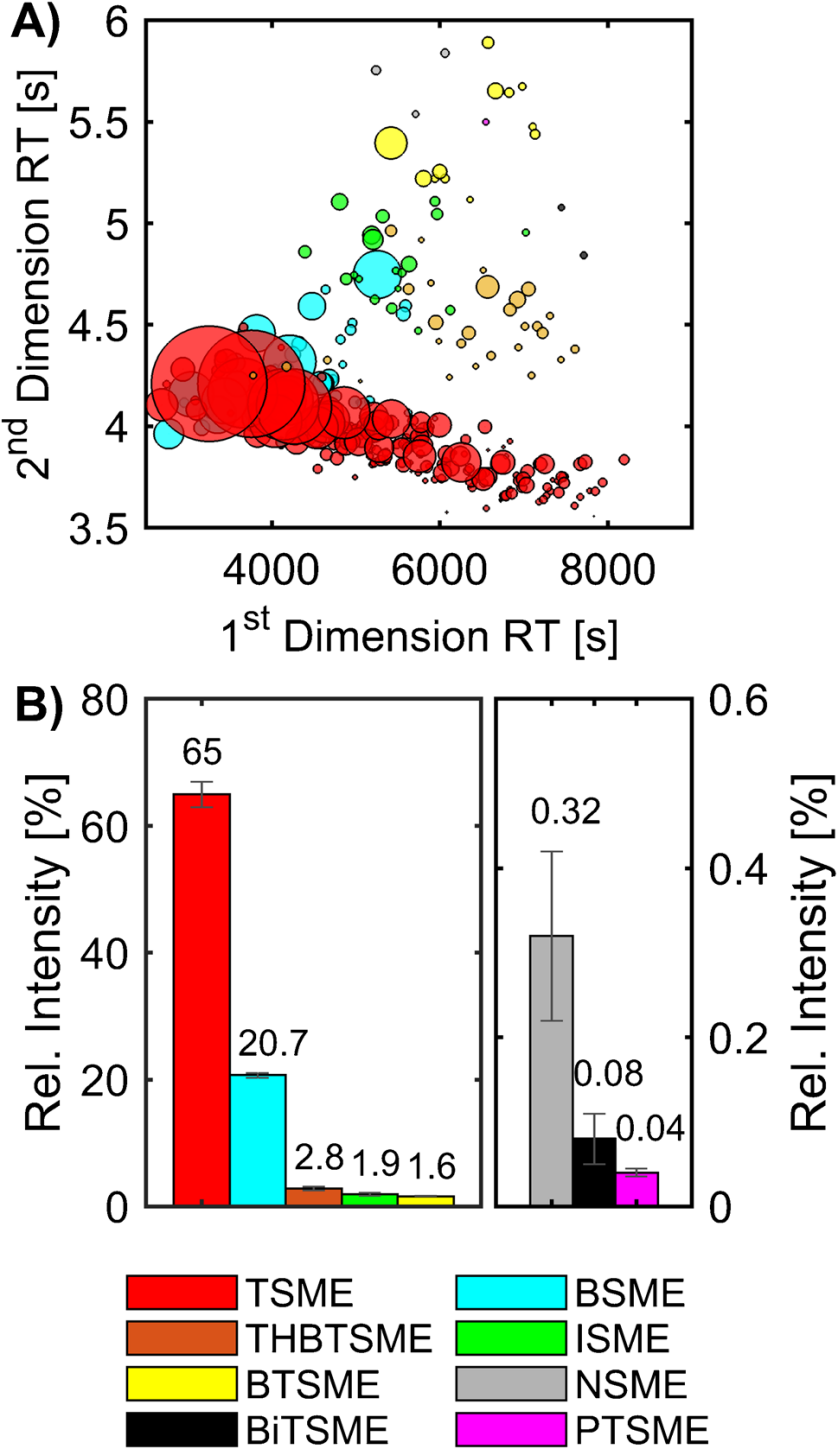
Overview of the chemical composition of the sodium bituminosulfonate API [16]. **A** Scatter plot of the GC × GC measurement for the detected sulfonates (sulfonate methyl esters (SMEs) with the core structural motive: thiophene (TSME), benzene (BSME), tetrahydrobenzothiophene (THBTSME), indane (ISME), benzothiophene (BTSME), naphthalene (NSME), bithiophene (BiTSME), and phenylthiophene (PTSME)) (altered from [16]). **B** Bar blot of their relative intensities with the standard error of the mean (*n* = 3)

Our previous publication on the chemical characterisation of SBS [16] presents the results of an online derivatisation measurement of SBS via GC × GC-HR-ToF–MS (Fig. 1A). We were able to identify eight groups of sulfonate methyl esters (SMEs) in the derivatised API; for seven, the core structural motive could be determined: thiophene (*TSME*), benzene (*BSME*), indane (*ISME*), benzothiophene (*BTSME*), naphthalene (*NSME*), bithiophene (*BiTSME*), and phenylthiophene (*PTSME*). The eighth compound class can tentatively be attributed to the tetrahydrobenzothiophene (*THBTSME*) core structure.

The applied method showed *TSME* and *BSME* as the most abundant compound classes (Fig. 1B). Based on the normalised detector response, they contribute to over 85% of the ion response. The results showed high experimental repeatability with relative standard deviations (RSDs) between 0.8 and 8.9% of the mean value and one with an RSD value of 30.2% for the second lowest abundant compound class (*BiTSME*). The order of class contribution for the sulfonates was comparable to the distillate precursors used for the sulfonation.

As a result, the previous study showed credible and repeatable in-depth untargeted results for the first time down to a molecular level for the complex API. Additionally, the use of the high-resolving GC × GC technique enables the application of a minimum extent of sample preparation.

Nevertheless, results might be biased due to sample preparation or the applied analytical methodology, and a simple plausibility check based on the exact identity of the precursor or literature might not be sufficient to meet the strict regulations of a pharmaceutical environment.

### Risk assessment of the analysis for sodium bituminosulfonate

The first step in evaluating the analytical procedure is to identify parameters that may affect the results. Therefore, the condition applied to the sample must be considered because the sample matrix can change qualitatively and quantitatively before (e.g. storage) and during the analytical workflow (sample preparation, measurement, and data handling). Therefore, the results may not reflect the original matrix but are altered by influencing factors, and a critical evaluation of the applied method is mandatory to ensure the reliability of the results. Risk assessment is a standard tool to evaluate numerous different procedures [19], and it will be recommended in the regulated pharmaceutical industry during the whole method development [14, 15]. It requires detailed information on the matrix and the applied analytical methodology. These requirements are met for target QC analysis with an established analytical process and in-depth knowledge of the matrix. Despite the absence of established procedures for complex mixtures and the lack of information on the matrix, it is compulsory to develop strategies that encompass risk assessment aspects to evaluate the reliability of the obtained results.

**Fig. 2:**
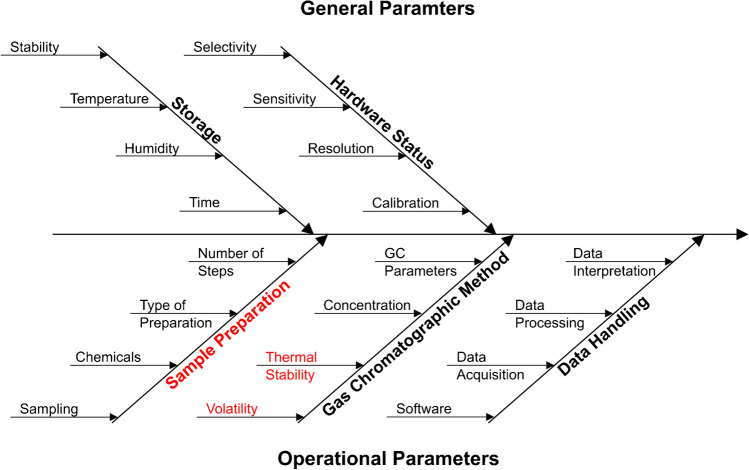
Ishikawa diagram of the estimated interferences of a GC × GC analysis for SBS. Divided into the adjustable operational parameter at the bottom and the general parameter at the top and ordered according to the time of their occurrence. The selected and further discussed parameters are highlighted in red

The parameters and their impact depend highly on the matrix and the used analytical methods. Therefore, the parameters must be identified, theoretically evaluated, and justified against the authorities [15]. Consequently, we followed the workflow from the arrival of the samples to the reporting of the results. We sorted the parameters into general or operational parameters, the assignment to a topic (storage, hardware status, sample preparation, gas chromatographic method, and data handling) and the time of their occurrence. As a result, we identified several possible major influencing parameters for the analysis of SBS via GC × GC and visualised them in an Ishikawa diagram (Fig. 2).

The diagram (Fig. 2) illustrates the amount of potential critical parameters that can only be rudimentarily evaluated due to the limited information on the matrix. We chose sample preparation, volatility (vapourability during the injection), and thermal stability (at the injection temperature) as, in our opinion, very critical parameters, as examples to evaluate their impact on the qualitative results and demonstrate the feasibility of such an approach. A quantitative evaluation and the determination of limit values, detection limits, or the performance of a total uncertainty budget was not the target of this study. However, these or similar evaluations would be essential to implement the investigated approach directly as a QC method in the pharmaceutical industry.

Besides an individual assessment of each parameter by reference methods, we also apply an alternative or complementary approach with the application of an orthogonal methodology. This evaluation sums up the effects of the operational parameters and the hardware status. Therefore, high similarities significantly increase the reliability of the results, but differences are difficult to assign to single parameters.

In summary, the risk assessment allows an overview of potential critical parameters. However, a limiting factor of such an approach is the lack of qualitative and quantitative information regarding the composition of the matrix. In particular, the missing quantitative information limits our evaluation. Nevertheless, a qualitative assessment can be carried out by selecting suitable reference methods.

### Extraction and derivatisation

The first part of the investigated analytical procedure is the sample preparation, which can drastically affect the quantitative and qualitative composition of the matrix [20] (Fig. 2 sample preparation). However, the limited information on the chemical composition of SBS and matrices similar to the API or its precursor distillate [21–24] hinders a comprehensive quantitative evaluation of these effects. For example, multiple compounds and classes may be present in the API besides sodium sulfonates, like unreacted starting material compounds and organic by-products [25]. Due to different physicochemical properties, compounds may behave differently during sample preparation. Still, it is necessary to address the entire matrix, including the low volatile and polar compounds such as sodium sulfonates. Consequently, the sample preparation should enable the applicability of a GC × GC analysis for sulfonates and maintain the original qualitative and quantitative composition.

**Fig. 3:**
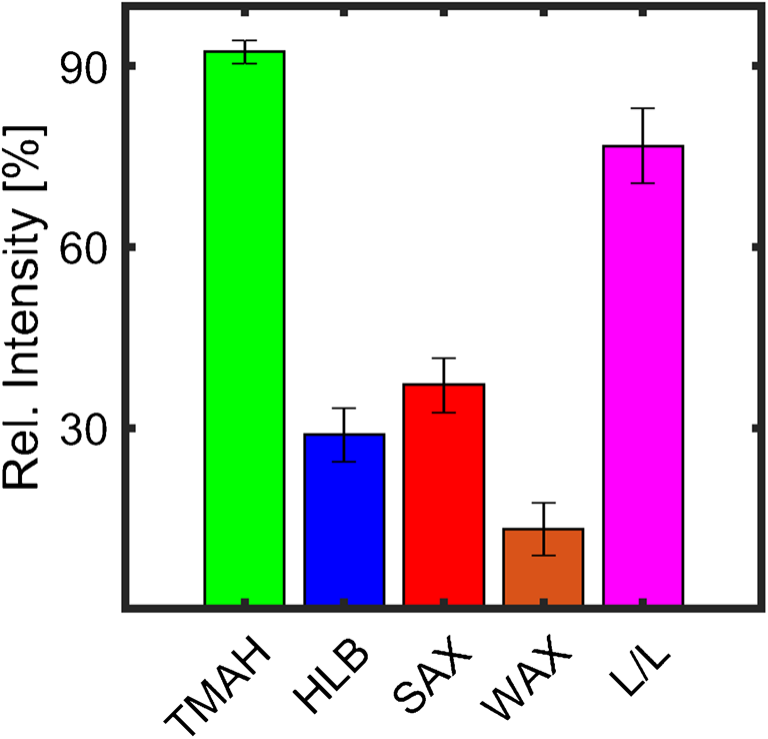
Bar plot of the relative intensities (to the overall ion count excluding the column bleed region) of sulfonate methyl esters (SMEs) in the GC × GC measurements and standard error of the mean (*n* = 3) obtained by the different extraction and derivatisation methods (online derivatisation via tetramethylammonium hydroxide (TMAH), offline derivatisation via trimethylsilyldiazomethane (TMSDAM) after solid-phase extraction with extraction sorbents hydrophilic–lipophilic balance (HLB), strong and weak anion exchanger (SAX and WAX), and liquid/liquid extraction (L/L))

Critical for the online derivatisation via TMAH are (1) the short reaction time that could be insufficient for the complete derivatisation of the sulfonates; (2) non-volatile compounds that are injected in the liner and thermally degrade; (3) the derivatisation agent TMAH, which could introduce side reactions, like thermochemolysis [26], and (4) discriminate against compounds. To investigate these possibilities, we used different SPEs and an L/L extraction followed by methylation with TMSDAM. The diazomethane derivative was chosen as the derivatisation agent because of its similarity to previously published methods used for GC analysis of sulfonates [27, 28]. This approach was designed to visualise the effect on the chemical profile of the derivatised API, in particular on the percentages of the SMEs as a possible indicator for the derivatisation efficiency (assuming a complete sulfonated matrix) (Fig. 3).

The five sample treatments resulted in significantly different relative intensities of SMEs, ranging from less than 20 to more than 80% (Fig. 3). In particular, the SPE methods resulted in a low percentage of SMEs. The remaining relative intensity is distributed to non-polar, non-sulfonated, and sulphur-containing aromatic compounds (e.g. thiophenes and benzothiophenes). This finding can be caused by multiple reasons, such as trapping or elution efficiency, which may result in an increased relative abundance of non-polar compounds. Additionally, a poor derivatisation efficiency could be present, but these should also be visible in the L/L extraction that used the same derivatising agent and showed a more similar percentage of SMEs as the TMAH online derivatisation. Additionally, the high reactivity in a sulfonation process [29] and the limited relative intensity of non-polar and non-sulfonated compounds after the online derivatisation and the L/L extraction indicate that only limited amounts of these compounds were initially present. However, TGA-MS of the API would allow determining the quantitative amount of these volatile organic compounds (see “Thermal behaviour of sulfonates”).

**Fig. 4:**
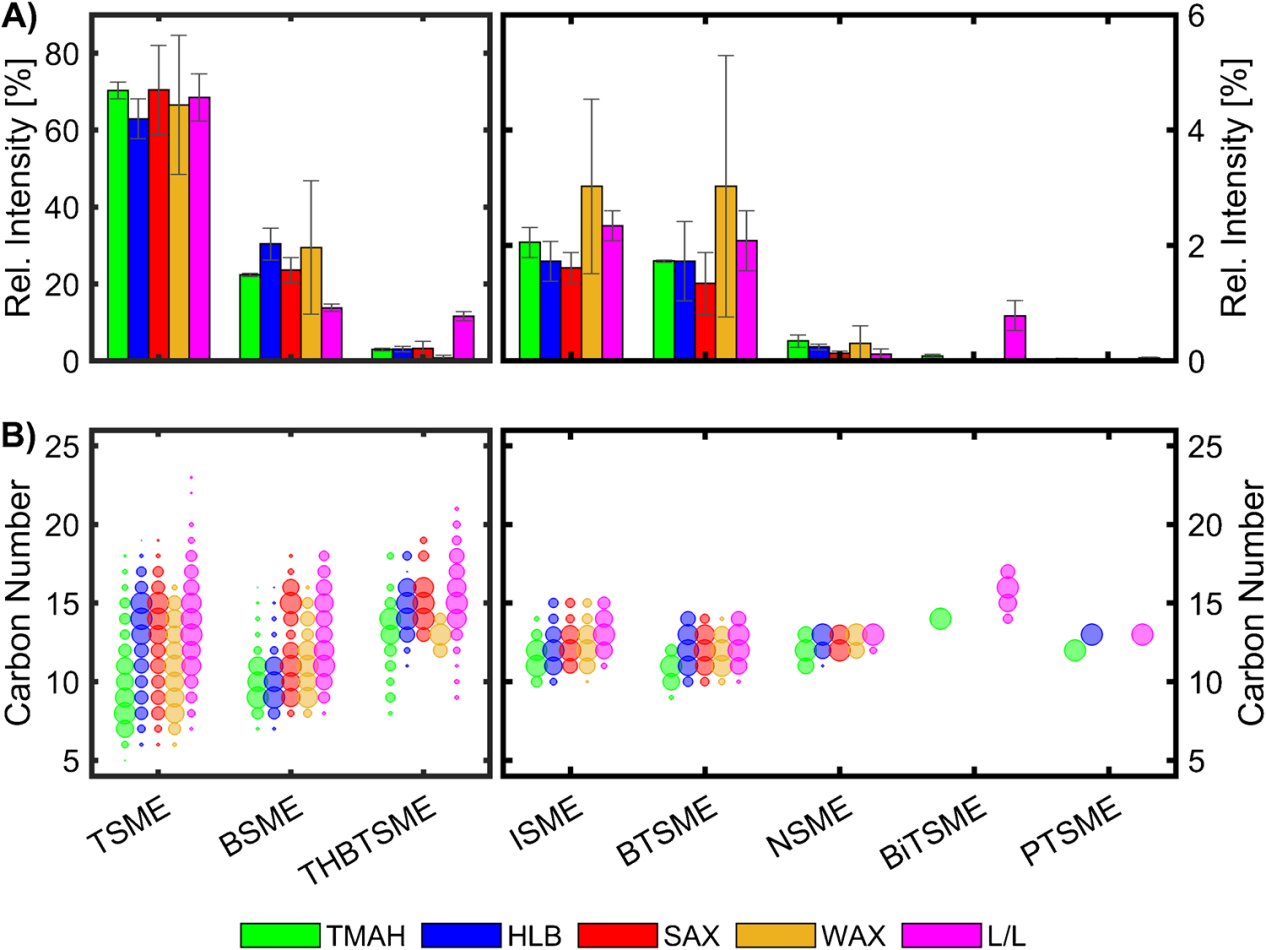
Influence of the extraction and derivatisation method (online derivatisation via tetramethylammonium hydroxide (TMAH), offline derivatisation via trimethylsilyldiazomethane (TMSDAM) after solid-phase extraction with extraction sorbents hydrophilic–lipophilic balance (HLB), strong and weak anion exchanger (SAX and WAX), and liquid/liquid extraction (L/L)) on the relative composition of the sulfonate methyl esters (SMEs). **A** Impact on the relative composition according to substance classes. Bar plot of the relative intensities and the standard error of the mean (*n* = 3) for the SMEs (sulfonate methyl esters with the core structural motive: thiophene (TSME), benzene (BSME), tetrahydrobenzothiophene (THBTSME), indane (ISME), benzothiophene (BTSME), naphthalene (NSME), bithiophene (BiTSME), and phenylthiophene (PTSME)) normalised to the overall percentage of sulfonates. **B** Impact on the relative composition according to carbon number. Aligned scatter plot of their carbon number distribution with bubble sizes reflecting their relative intensity normalised to the most intense peak in the compound class of the sample preparation method

Besides the SMEs and the sulphur aromatic compounds, none of the offline sample preparation methods presented compounds that could be hydrolysed by TMAH or additional polar compounds or compound classes like disulfonated arenes. These findings indicate that TMAH did not induce side reactions during the online derivatisation and does also not discriminate major constituents of the matrix compared to the reference methods. In the next step, we investigated the influence of the preparation method on the relative composition (Fig. 4).

One of the most apparent differences is the variance of the standard errors between the sample preparations (Fig. 4A). In particular, the SPE with WAX cartridge shows high variances and even the other offline sample preparations with smaller variations, of which the L/L extraction shows the smallest, do not provide a similar precision as the online derivatisation with TMAH.

The distribution of the sulfonated compound classes is more comparable. However, there are significant differences in their percentages. In particular, the L/L extraction shows a higher percentage of *THBTSME* and *BiTSME* in parallel with a lower value for *BSME*. This results from the different physicochemical interactions and affinities (e.g. stationary phases in SPE and distribution between the organic and aqueous phases in the L/L extraction). While the SPE-based methods target the sulfonate anion (functional group), the L/L extraction with toluene interacts with the aromatic core structural motive. This affects and shifts the chemical composition after the L/L extraction compared to the SPE and online derivatisation methods to larger and more non-polar compounds.

This is also visible in the carbon number distribution (Fig. 4B). Compared to the online derivatisation, all extraction methods did not detect non-alkylated thiophene and benzenesulfonate methyl ester, but particularly, the L/L extraction detected larger compounds. Simultaneously, it shows a shift in the intensity distribution to higher carbon numbers. This carbon number distribution is close to the distribution in the starting material (supplemental information (SI): Fig S1). However, the starting material did not show aromatic compounds with carbon numbers higher than 15 and included isomers that are completely alkylated and cannot form sulfonates. Therefore, the distribution of the online derivatisation is closer to our expected distribution, and the shift in the L/L extraction is caused by the extraction procedure, but it is beneficial to complement the carbon profile and to visualise the limitation of the online derivatisation method.

In summary, online derivatisation shows the highest yield of SMEs, the best repeatability, and the most minor influence on the carbon number distribution. In addition, it qualitatively covers most of the compounds detected with the alternative sample preparations with a minimum instrumental effort. However, the comparison shows the significant effect of the sample preparation on the chemical composition. These results would indicate the necessity for multiple standards from numerous compound classes and with different carbon numbers to establish a valid quantitative evaluation. However, all findings are limited to the accessibility of the matrix using GC. As a result, the vapourability of the API must be evaluated with additional methods.

### Thermal behaviour of sulfonates

After the sample preparation, the injection temperature (350 °C) could be another critical parameter (Fig. 2 volatility and thermal stability). Thermal breakdown and conversion during the injection and lack of volatility would limit the reliable applicability of the method and could corrupt the qualitative information. For the investigation of the general thermal behaviour of the matrix, TGA was used to monitor the temperature-resolved mass loss and, in parallel, chemical speciation of the evolving gas mixture by MS (TGA-MS). Although TGA-MS is an approved tool to address and investigate thermal and pyrolytic processes [30, 31], the target of this analysis is to evaluate the quantitative accessibility of the sample (mass loss and thermal stability < 350 °C). A detailed discussion of the underlying degradation reactions would go beyond the scope of this manuscript. We have therefore provided appropriate references.

**Fig. 5:**
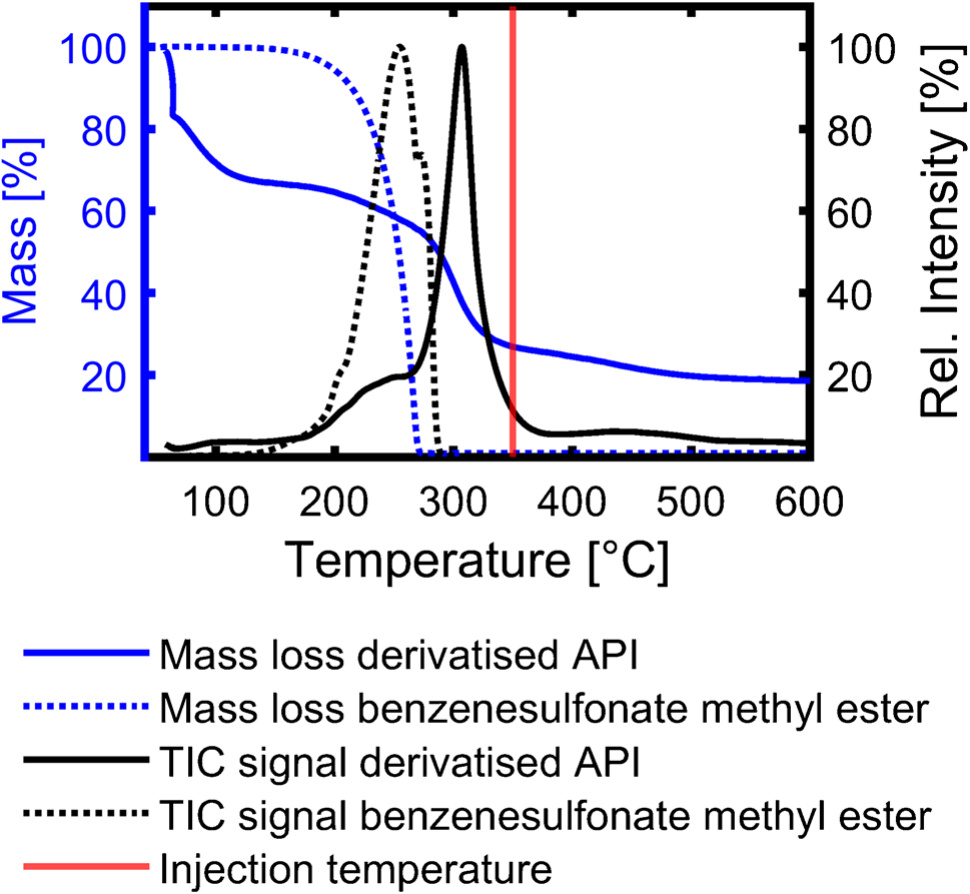
Thermogravimetric analysis of the derivatised API and benzenesulfonate methyl ester. Total ion current and mass loss of the derivatised API and the benzenesulfonate (zoomed below 600 °C). The applied injection temperature of the GC method (350 °C) is indicated as a reference. The mass spectra tables are in supplemental information (Table S1 and S4)

Because of the high physicochemical complexity of SBS, we also investigated commercially available individual sulfonates (benzenesulfonate methyl ester, sodium benzenesulfonate, and benzenesulfonic acid). Benzenesulfonate is a constituent of the API, and the derivatives should indicate the thermal behaviour of the non-extracted API (sodium sulfonate), the extracted API (sulfonic acid), and the derivatised API (sulfonate methyl ester). Other possible more abundant compounds, such as sulfonated and alkylated thiophenes, were not commercially available. Due to the flat temperature gradient of a TGA and the volatility of the derivatisation agent, it is technically not possible to simulate the TMAH online derivatisation via TGA-MS. Therefore, the TMSDAM derivatised L/L extracted served as the basis for the investigation (Fig. 5). The derivatisation products should be similar. Possible differences and complications arising from this are discussed below.

**Fig. 6:**
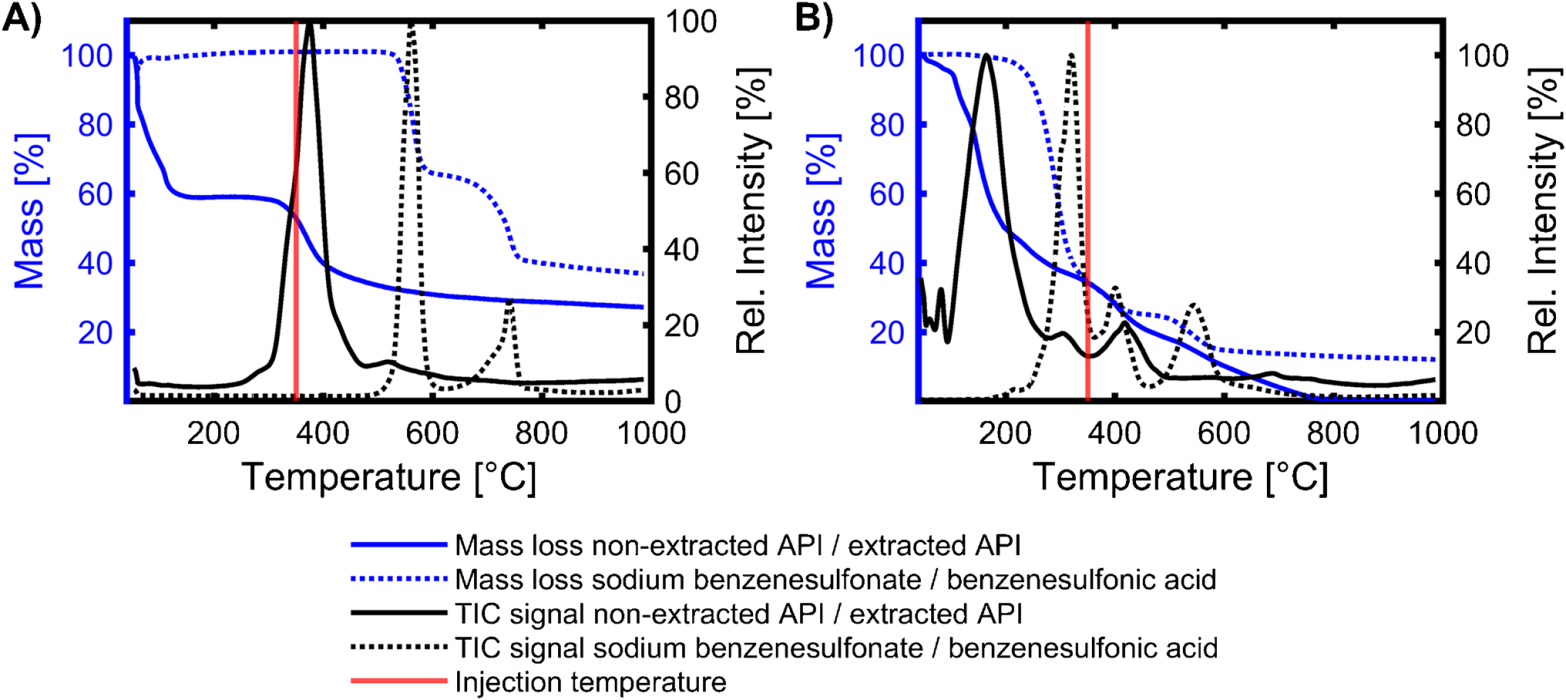
Thermogravimetric analysis of the non-extracted API and the extracted API. **A** Total ion current and mass loss of the non-extracted API together with sodium benzenesulfonate and **B** the extracted API with benzenesulfonic acid. The applied injection temperature of the GC method (350 °C) is indicated as a reference. The mass spectra tables are in supplemental information (Table S2, S3, S5, and S6)

The first mass loss up to 100 °C (about 35 w%) of the derivatised API (Fig. 5) resulted from residues of the extraction (toluene), the solvents (methanol and acetone), and the volatile derivatisation agent (TMSDAM). Between 200 and 350 °C, an additional 40 w% of the derivatised API evaporates. This intermediate temperature step should correspond to the compounds also seen in the GC × GC analysis. Consequently, the acquired mass spectrum for the TGA-MS shows mass fragment ions of the same aromatic core structures, which could also be found in the GC × GC analysis (Table S4). However, even at 600 °C, about ~ 20 w% remain in the crucible. This may be caused by pyrolytic residue formed by the thermal degradation of non-volatile and polar constituents, such as disulfonates, sulfonic acids, or sodium sulfonate species, causing stable char [32] or inorganic oxides/salts. Also, derivatisation artefacts like the formation of siloxanes might be possible [33]. In contrast to the derivatised API, the corresponding single compound benzenesulfonate methyl ester showed complete evaporation (< 300 °C), and the mass spectrum shows only *m/z* signals associated with the EI fragmentation of the methyl ester (SI: Table S1). This indicates the general vapourability of the derivatised API. The shift in the evaporation temperature from benzenesulfonate methyl ester to the derivatised API can be explained by the alkylation and the presence of several compound classes. For a further evaluation of the remaining 20 w% of the derivatised API, which seems to be discriminated by the applied GC × GC method, we also investigated the thermal behaviour of the different precursors, which might be present due to an insufficient derivatisation process (Fig. 6).

The first mass loss event in the non-extracted API (sodium salt) is caused by the evaporation of water (about 40 w%), and temperatures above 300 °C were required to induce a second mass loss event, where the evaporating gases are detected by the MS (Fig. 6A). The missing TIC signal below this temperature indicates the absence of quantitative amounts of volatile organic compounds (e.g. remaining from the precursor distillate). In addition, at temperatures above 300 °C, the mass loss may result from thermal breakdown of the sample. This could be seen in the analysis of the corresponding single compound sodium benzenesulfonate. Here, at temperatures above 500 °C, the mass spectrum (SI: Table S2) indicates the presence of diphenyl sulphide, a known thermal degradation product of benzenesulfonates [29]. At higher temperatures (> 650 °C), SO_2_ and CO_2_ were formed. At the end of the TGA (1000 °C), more than 25 w% of the non-extracted API and sodium benzenesulfonate remanined in the crucible.

The extracted API shows the first mass loss and corresponding MS signal between 100 and 250 °C (Fig. 6B). Besides this event, the extracted API show a complete mass loss (< 800 °C), indicating the absence of inorganic sodium salts. The TGA analysis of the single compound benzenesulfonic acid shows two mass loss events with the same mass spectra between 250–450 °C (SI: Table S3). They indicate the evaporation of benzenesulfonic acid in parallel to the thermal degradation product diphenyl sulfone [34]. Conclusively, the benzenesulfonic acids show thermal instability below the injection temperatures, and the shift to even lower temperatures in the extracted API may indicate a higher thermal instability of the predominant sulfonated sulphur arenes.

Our study has shown that the thermal processes during injection are a critical factor of the method and must be evaluated. Through targeted reference measurements on a TGA system using matrix and single substances, we were able to prove that we can reproduce the quantitative information and that no discrimination occurs. After a mandatory derivatisation of the matrix, no degradation or other reactions could be detected that would falsify the qualitative information. An initially substantial non-volatile residue of 20% might be due to the derivatisation with TMSDAM, which had to be used instead of TMAH due to the experimental setup.

### Cross-verification by Fourier transform ion cyclotron resonance mass spectrometry

The measurements in the previous chapters showed the impact of the sample treatment on the matrix, the inter-dependence on each other, and a possible physicochemical limitation of the applied gas chromatographic technique. This limits the significance of evaluating individual parameters. Therefore, the performance of a suitable alternative analytical method should address the general applicability of the GC × GC analysis (Fig. 2 gas chromatographic method). Conclusively, possible non-volatile and polar compounds should be targeted, preferably without sample preparation, to verify the GC × GC results. Here, DI ESI( −) FT-ICR MS presents itself as a viable method for cross-verification. The high sensitivity and soft ionisation properties of ESI( −) for acidic and polar compounds, such as the sodium sulfonates in the API, combined with the excellent mass resolution and accuracy, allow the differentiation between species and the determination of the elemental composition [35–37]. Without additional sample preparation, the diluted sample is measured directly from the solution, limiting thermal stress on the compounds. Therefore, it is a suitable method for the qualitative evaluation of polar compounds in SBS and for complementing the verification of the GC × GC results by selectively targeting the compounds challenging to gas phase analysis. However, direct infusion mass spectrometry without any chromatographic preparation is not able to address individual isomers, which might be a prerequisite for the establishment of a QC method.

**Fig. 7:**
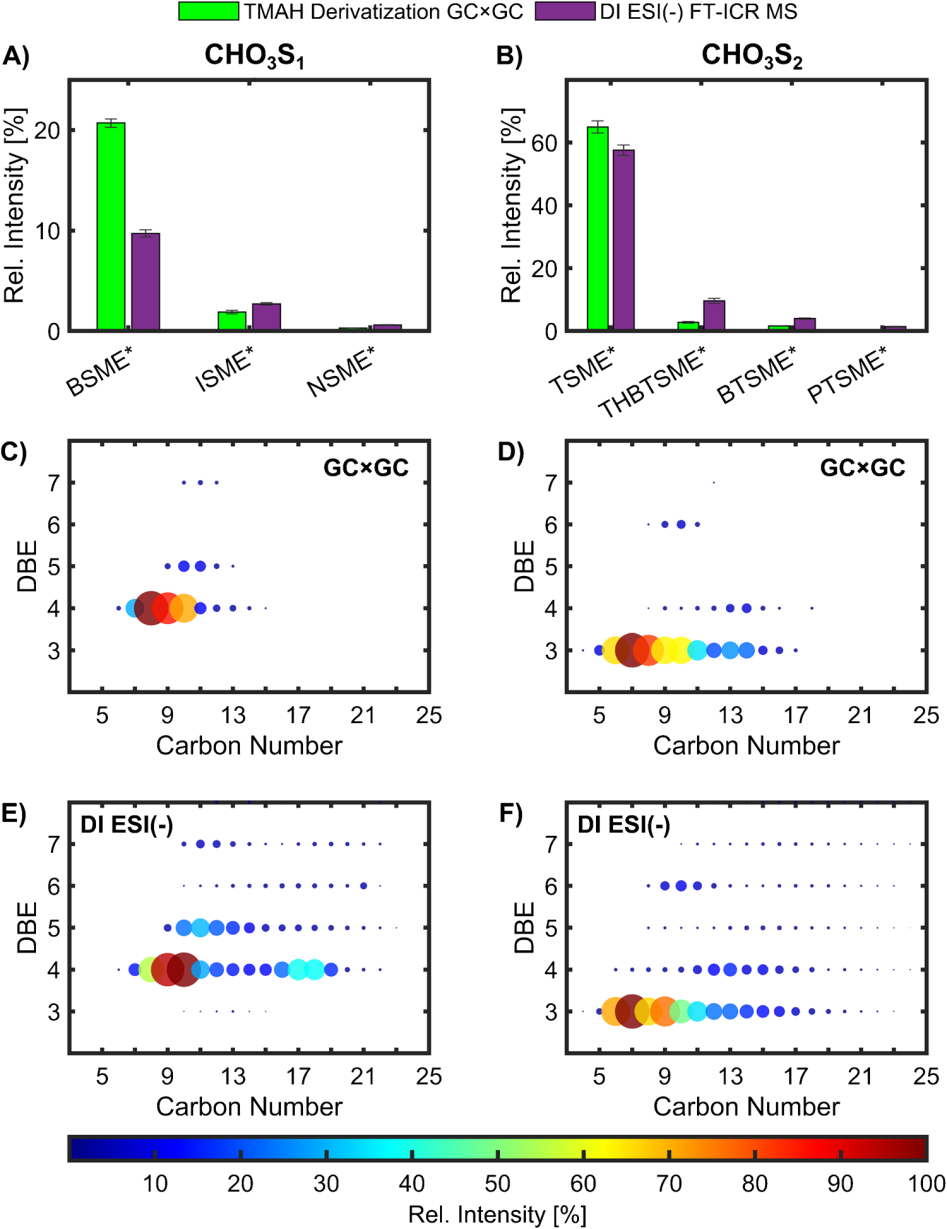
Comparison of the TMAH online derivatised GC × GC and DI ESI( −) FT-ICR MS results. **A** Bar plot of the relative intensities and the standard error of the mean (*n* = 3) on sulfonated hydrocarbons (CHO_3_S_1_) (sulfonate methyl esters (SMEs) with the core structural motive: benzene (BSME), indane (ISME), and naphthalene (NSME); *detected as sulfonate in the FT-ICR MS) and **B** sulfonated sulphur arenes (CHO_3_S_2_) (sulfonate methyl esters (SMEs) with the core structural motive: thiophene (TSME), tetrahydrobenzothiophene (THBTSME), benzothiophene (BTSME), and phenylthiophene (PTSME); *detected as sulfonate in the FT-ICR MS) of both techniques. **C**,** E** DBE versus carbon number plot of the detected sulfonated hydrocarbons and **D**,** F** sulphur species **C**, **D** detected as SMEs by GC × GC (the carbon atom from the methylation was removed) and **E**, **F** sulfonate anion by DI ESI( −) FT-ICR MS zoomed to the double bound equivalent (DBE) 2–8 and C3–25 (complete DBE vs C plot in SI: Fig S2). Bubble size and colour represent the relative intensity normalised to the most abundant species in the plot

Due to the different nature of the two analytical systems, the resulting data are initially not comparable and must be first translated to a common format (e.g. compound classes and elemental composition based on MS information) in order to create a comparable data basis for the evaluation of the data. Therefore, the relative intensity of compounds with the same accurate masses was calculated by adding the summed ion count of the isomers and dividing by the overall summed ion count (excluding the column bleed region). In addition, the species information from the SMEs in the GC × GC-HR-ToF–MS and the sulfonate anions in the DI ESI( −) FT-ICR MS must be unified. Therefore, GC × GC data are processed as sulfonic acid equivalents and not as derivatised species. For the GC × GC measurements, the transfer was achieved by reducing the determined ionic sum formulae by CH_2_ to achieve the non-methylated molecular sum formulae. Analogous to this, the determined anionic sum formulae identified by DI ESI( −) were converted into their corresponding neutral molecular sum formulae. The results of both methods are compared in Fig. 7.

Although the summed relative intensities of the compound classes are different, they showed a similar distribution for the most abundant compound classes, with sulfonated thiophenes accounting for more than 50% of the relative intensity for both techniques featuring different physical principles (Fig. 7A and B). The seven sulfonated compound classes with none or one sulphur atom in the core structural motive detected by GC × GC account for more than 85% of the relative intensity in DI ESI( −) FT-ICR MS. However, DI ESI( −) can access an additional 12% (Table S7). Here, the majority (8.4%) is composed of CHO_3_S_3_ species (7.3%) and CHO_3_S_4_ species (1.1%). However, they include many low-intense signals (CHO_3_S_3_, 104 sum formulae, all below 1% and 84 of them below 0.1% relative to the summed ion count) that accumulate to these relatively high percentages. For the GC × GC-HR-ToF–MS, the masses are divided in the multiple isomers, and for the low intense FT-ICR MS signals, they might be below the detection limit. In addition, the low intensity or absence of the precursors and the distillation phase of the distillate starting material indicate a low concentration of these species.

The relative intensity of all sulfonates detected by DI ESI( −) FT-ICR MS accumulates over 95%. Therefore, no significant polar compounds and compound classes are present besides sulfonates, and the most abundant classes (> 2% relative intensity in the DI ESI( −) (SI: Table S8)) are detected by both methods. However, the trends between the measuring techniques are visible. While the GC × GC showed a higher relative intensity of one-ring aromatic species (*TSME* and *BSME*), higher aromatic and heavier species are more prominent in the DI ESI( −) FT-ICR MS for sulfonated hydrocarbons and sulphur arenes.

A similar difference is visualised in the DBE vs. carbon number plots (Fig. 7C–E). These plots represent the compound classes with the same heteroatom composition distributed in their unsaturation with the DBE as the integer value [16] and the carbon number to visualise the alkylation. Here, the DI ESI( −) FT-ICR MS presented more peaks (broader coverage of the alkylation), but the relative intensity of the species not present in the GC × GC analysis is comparably low. The species not covered by the seven compound classes accumulate to 3.3% of the relative intensity in the DI ESI( −) measurements. However, the increasing intensity for DBE 4 in DI ESI( −) with a carbon number > 16 (2.6% relative intensity in the DI ESI( −)) indicates a potential additional compound class with the same elemental composition as sulfonated benzenes above the volatility limit of the GC × GC. In general, the difference in the alkylation range and compound class distribution between these two techniques may be induced by the additional separation of isomers in GC × GC that are summed up by the DI ESI( −) FT-ICR MS, in addition to other factors like the selectivity of ESI, the sensitivity of FT-ICR MS for higher *m/z* values, and limited volatility.

In summary, both methods show significant differences, but most of them could be associated with the sensitivities and the affinities of the methods. Consequently, the non-negligible similarities of the orthogonal methods support the plausibility of the qualitative results. Together with the individual investigations of the other critical parameters, identified in the risk assessment (sample preparation and thermal stability), and their support of the chemical profile, this comparison shows the suitability of the online derivatisation GC × GC-HR-ToF–MS to access qualitatively a decisive and significant part of the API.

## Conclusion

In this work, the risk assessment was the base for the identification and evaluation of critical parameters for a qualitative untargeted analysis of a complex API. Exemplarily, we showed the possible distorting influence effect of the sample preparation and injection temperature on the qualitative composition of SBS. The sample preparation has a significant influence on the qualitative signature, but online derivatisation reflects a balanced picture of the matrix without significantly discriminating certain chemical groups. Furthermore, TGA-MS experiments indicate that the qualitative data is not affected by the injection temperature and residuals measured above 350 °C result from derivatisation fragments of the TMSDAM.

In addition to an individual assessment of the risk factors, we were also able to target the physicochemical limitations of the method holistically due to the application of an orthogonality technique. Here, DI ESI( −) FT-ICR MS allowed us to evaluate the combined effects from most of the methodological parameters identified in the risk assessment. As a result, we could show that the two orthogonal methods largely produce the same qualitative fingerprint of the matrix. In addition, we did not find physiochemical limitations with regard to the matrix under investigation for the selected GC × GC method.

In summary, it can be concluded that the selected method of online derivatisation GC × GC-HR-ToF–MS is capable of revealing the chemical identity of the matrix. Such approaches might be very challenging and demanding but open the possibility of incorporating also state-of-the-art and complex methodologies in controlled environments and to justify the further usage of the data as basis for further quantitative analysis. Other complex drugs, particularly NBCDs, might present similar challenges for the analytical procedures and would benefit from a verified chemical profile from a comprehensive untargeted analysis.

### Supplementary Information

Below is the link to the electronic supplementary material.

Supplementary file1 (DOCX 320 KB)
