# Supplementary material for: Applying a risk assessment guided evaluation for verifying comprehensive two-dimensional gas chromatography to analyse complex pharmaceuticals
Source: Anal Bioanal Chem. 2023 Dec 21;416(4):1033–45. doi: 10.1007/s00216-023-05093-9 (PMC10800299; doi:10.1007/s00216-023-05093-9)
Supplement: Supplementary file 1 — Supplementary file1 (DOCX 320 KB) [file 216_2023_5093_MOESM1_ESM.docx]

**Supplemental Information**

**Applying a risk assessment guided evaluation for verifying comprehensive two-‑dimensional gas chromatography to analyse complex pharmaceuticals**

Lukas Schwalb^1,2^; Ole Tiemann^1,3^; Uwe Käfer^1,2,4^; Christopher Paul Rüger^1,3^; Thomas Gröger^2,*^; Ralf Zimmermann^1,2,3^

^1^ Joint Mass Spectrometry Centre (JMSC), Chair of Analytical Chemistry, University of Rostock, Rostock, Germany

^2^ Joint Mass Spectrometry Centre (JMSC), Cooperation Group “Comprehensive Molecular Analytics” (CMA), Helmholtz Zentrum München GmbH, German Research Center for Environmental Health, Neuherberg, Germany

^3^ Department Life, Light & Matter (LLM), University of Rostock, Rostock, Germany

^4^ now at: Leibniz-Institute of Tropospheric Research (TROPOS), Leipzig, Germany

*Corresponding Author: Thomas Gröger <thomas.groeger@helmholtz-munich.de>

**Abbreviations**

API Active pharmaceutical ingredient

SBS Sodium bituminosulfonate

GC×GC Comprehensive two-dimensional gas chromatography

HR-ToF-MS High resolution time-of-flight mass spectrometer

TGA Thermogravimetric analysis

DI ESI(-) FT-ICR MS Direct infusion negative electrospray ionisation Fourier transform ion cyclotron resonance mass spectrometry

QC Quality control

ICH International Council for Harmonization

NBCD Non-biological complex drug

TMAH Tetramethylammonium hydroxide

TMSDAM Trimethylsilyl diazomethane

SPE Solid phase extraction

WAX Weak anion exchanger

SAX Strong anion exchanger

HLB hydrophilic-lipophilic balance

L/L Liquid/liquid (extraction)

SME Sulfonate methyl ester (R-SO_3_-CH_3_)

TSME Thiophenesulfonate methyl ester

BSME Benzenesulfonate methyl ester

THBTSME Tetrahydrobenzothiophenesulfonate methyl ester

ISME Indansulfonate methyl ester

BTSME Benzothiophenesulfonate methyl ester

NSME Naphthalenesulfonate methyl ester

BiSME Bithiophenesulfonate methyl ester

PTSME Phenylthiophenesulfonate methyl ester

SI Supplemental information

DBE Double bound equivalent


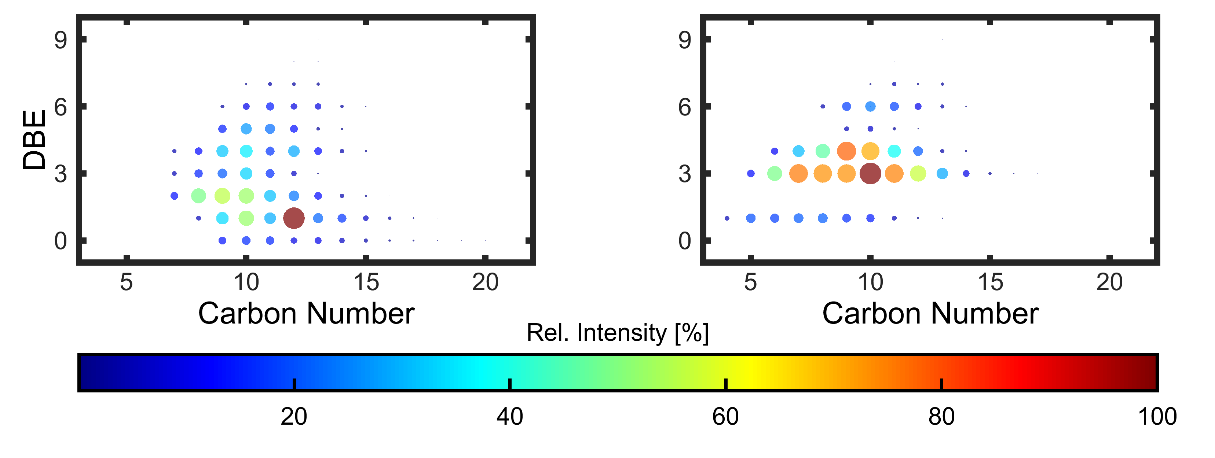


Fig. S1 DBE vs. Carbon number plot of the light distillate (starting material) with CH (left) and the CHS elemental composition (right). Bubble size and colour representing the relative intensity normalized to the most abundant species in the plot.


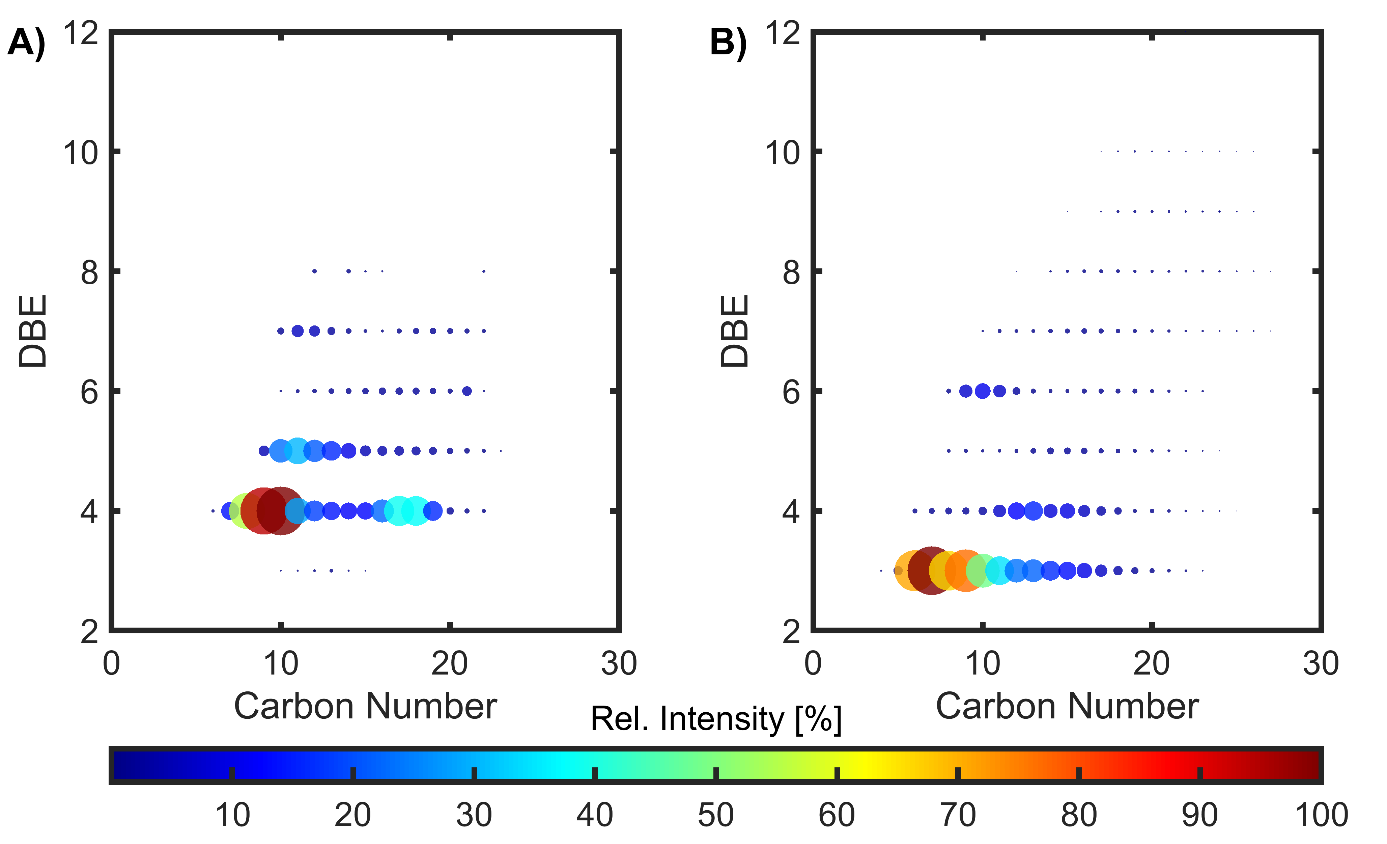


Fig. S2 Complete DBE vs. Carbon number plot for the DI ESI(-) FT-ICR MS measurement of the sulfonated hydrocarbons and (left): sulphur species (right): Bubble size and colour representing the relative intensity normalized to the most abundant species in the plot.

Table S1 Assigned m/z values of the mass loss event in the TGA-MS of the benzenesulfonic acid methyl ester (Fig 5 **B**). Temperature range 150-300 °C.

| Assigned Exact Mass  [Da] | Relative Abundance  [%] | Mass  Error [ppm] | C | H | N | O | S | DBE  Value |
| --- | --- | --- | --- | --- | --- | --- | --- | --- |
| 50.01528 | 12.05 | 4.3 | 4 | 2 | 0 | 0 | 0 | 4 |
| 51.02297 | 30.44 | 1.6 | 4 | 3 | 0 | 0 | 0 | 3.5 |
| 65.03823 | 5.85 | -4.8 | 5 | 5 | 0 | 0 | 0 | 3.5 |
| 77.03803 | 100.00 | -6.6 | 6 | 5 | 0 | 0 | 0 | 4.5 |
| 78.04567 | 15.72 | -8.9 | 6 | 6 | 0 | 0 | 0 | 4 |
| 78.9847 | 11.18 | -1.2 | 1 | 3 | 0 | 2 | 1 | 0.5 |
| 141.00027 | 28.79 | -1.2 | 6 | 5 | 0 | 2 | 1 | 4.5 |
| 172.01849 | 19.31 | -2.0 | 7 | 8 | 0 | 3 | 1 | 4 |

Table S2 Assigned m/z values of the first mass loss event in the TGA-MS of the sodium benzenesulfonate (Fig 6 **B**). Temperature range 500-600 °C.

| Assigned Exact Mass  [Da] | Relative Abundance  [%] | Mass  Error [ppm] | C | H | N | O | S | DBE  value |
| --- | --- | --- | --- | --- | --- | --- | --- | --- |
| 42.00882 | 10.11 | -27.6 | 2 | 2 | 0 | 1 | 0 | 2 |
| 43.98939 | 39.29 | 3.3 | 1 | 0 | 0 | 2 | 0 | 2 |
| 47.96643 | 17.53 | 0.6 | 0 | 0 | 0 | 1 | 1 | 1 |
| 50.01529 | 16.16 | 4.5 | 4 | 2 | 0 | 0 | 0 | 4 |
| 51.02311 | 39.45 | 4.3 | 4 | 3 | 0 | 0 | 0 | 3.5 |
| 52.03075 | 7.48 | 0.7 | 4 | 4 | 0 | 0 | 0 | 3 |
| 63.02167 | 7.02 | -19.3 | 5 | 3 | 0 | 0 | 0 | 4.5 |
| 63.96141 | 39.03 | 1.5 | 0 | 0 | 0 | 2 | 1 | 1 |
| 65.03855 | 15.74 | 0.2 | 5 | 5 | 0 | 0 | 0 | 3.5 |
| 68.97918 | 11.41 | -1.9 | 3 | 1 | 0 | 0 | 1 | 3.5 |
| 74.01467 | 6.10 | -5.3 | 6 | 2 | 0 | 0 | 0 | 6 |
| 77.03848 | 29.11 | -0.8 | 6 | 5 | 0 | 0 | 0 | 4.5 |
| 78.04618 | 31.11 | -2.4 | 6 | 6 | 0 | 0 | 0 | 4 |
| 92.01703 | 10.82 | 6.6 | 2 | 6 | 1 | 1 | 1 | 0.5 |
| 109.0101 | 8.39 | -4.7 | 6 | 5 | 0 | 0 | 1 | 4.5 |
| 115.05443 | 5.64 | 2.1 | 9 | 7 | 0 | 0 | 0 | 6.5 |
| 139.05437 | 6.99 | 1.3 | 11 | 7 | 0 | 0 | 0 | 8.5 |
| 152.06157 | 16.01 | -2.9 | 12 | 8 | 0 | 0 | 0 | 9 |
| 153.06706 | 7.04 | -18.2 | 12 | 9 | 0 | 0 | 0 | 8.5 |
| 154.07799 | 6.14 | 2.1 | 12 | 10 | 0 | 0 | 0 | 8 |
| 171.02632 | 8.91 | 0.3 | 11 | 7 | 0 | 0 | 1 | 8.5 |
| 184.03432 | 53.92 | 1.3 | 12 | 8 | 0 | 0 | 1 | 9 |
| 185.04114 | 84.01 | -4.2 | 12 | 9 | 0 | 0 | 1 | 8.5 |
| 186.05039 | 100.00 | 3.5 | 12 | 10 | 0 | 0 | 1 | 8 |
| 187.05355 | 13.83 | -3.4 | 15 | 7 | 0 | 0 | 0 | 12.5 |
| 262.08109 | 8.36 | 0.2 | 18 | 14 | 0 | 0 | 1 | 12 |

Table S3 Assigned m/z values of the first two mass loss events in the TGA-MS of the benzenesulfonic acid (Fig 6 **D**). Temperature range 250-450 °C

| Assigned Exact Mass  [Da] | Relative Intensity  [%] | Mass  Error  [ppm] | C | H | N | O | S | DBE  value |
| --- | --- | --- | --- | --- | --- | --- | --- | --- |
| 50.01501 | 28.89 | -1.1 | 4 | 2 | 0 | 0 | 0 | 4 |
| 51.02273 | 59.59 | -3.1 | 4 | 3 | 0 | 0 | 0 | 3.5 |
| 52.03075 | 15.37 | 0.7 | 4 | 4 | 0 | 0 | 0 | 3 |
| 63.02296 | 5.43 | 1.1 | 5 | 3 | 0 | 0 | 0 | 4.5 |
| 63.96128 | 73.01 | -0.6 | 0 | 0 | 0 | 2 | 1 | 1 |
| 65.03869 | 13.68 | 2.3 | 5 | 5 | 0 | 0 | 0 | 3.5 |
| 66.0464 | 6.08 | 0.5 | 5 | 6 | 0 | 0 | 0 | 3 |
| 74.01457 | 10.00 | -6.7 | 6 | 2 | 0 | 0 | 0 | 6 |
| 76.0306 | 9.20 | -1.5 | 6 | 4 | 0 | 0 | 0 | 5 |
| 77.03874 | 93.49 | 2.6 | 6 | 5 | 0 | 0 | 0 | 4.5 |
| 78.04665 | 66.60 | 3.7 | 6 | 6 | 0 | 0 | 0 | 4 |
| 94.04086 | 23.35 | -4.5 | 6 | 6 | 0 | 1 | 0 | 4 |
| 97.01052 | 13.84 | -0.9 | 5 | 5 | 0 | 0 | 1 | 3.5 |
| 125.00532 | 100.00 | -1.6 | 6 | 5 | 0 | 1 | 1 | 4.5 |
| 126.00933 | 7.38 | -5.2 | 9 | 2 | 0 | 1 | 0 | 9 |
| 152.06179 | 5.33 | -1.5 | 12 | 8 | 0 | 0 | 0 | 9 |
| 158.00366 | 16.61 | 3.0 | 6 | 6 | 0 | 3 | 1 | 4 |
| 218.03989 | 8.10 | 1.5 | 12 | # | 0 | 2 | 1 | 8 |

Table S4 Assigned m/z values of the mass loss event between 200-350 °C in the TGA-MS of the derivatized L/L extract (Fig 5 **A** and S1 **A**).

| Assigned Exact Mass  [Da] | Relative Intensity  [%] | Mass  Error  [ppm] | C | H | N | O | S | DBE  value |
| --- | --- | --- | --- | --- | --- | --- | --- | --- |
| 40.03108 | 7.00 | 9.1 | 3 | 4 | 0 | 0 | 0 | 2 |
| 41.03881 | 31.23 | 6.6 | 3 | 5 | 0 | 0 | 0 | 1.5 |
| 42.04624 | 3.32 | -3.0 | 3 | 6 | 0 | 0 | 0 | 1 |
| 43.0177 | 6.14 | -2.4 | 2 | 3 | 0 | 1 | 0 | 1.5 |
| 43.05435 | 10.84 | 3.7 | 3 | 7 | 0 | 0 | 0 | 0.5 |
| 43.9893 | 34.91 | 1.3 | 1 | 0 | 0 | 2 | 0 | 2 |
| 44.02583 | 3.10 | 4.6 | 2 | 4 | 0 | 1 | 0 | 1 |
| 44.97925 | 28.59 | -1.4 | 1 | 1 | 0 | 0 | 1 | 1.5 |
| 45.0338 | 16.54 | 7.7 | 2 | 5 | 0 | 1 | 0 | 0.5 |
| 45.98706 | 3.80 | -1.7 | 1 | 2 | 0 | 0 | 1 | 1 |
| 46.04129 | 4.14 | 0.2 | 2 | 6 | 0 | 1 | 0 | 0 |
| 46.99457 | 15.30 | -8.3 | 1 | 3 | 0 | 0 | 1 | 0.5 |
| 47.96626 | 29.15 | -2.9 | 0 | 0 | 0 | 1 | 1 | 1 |
| 48.00267 | 5.74 | -2.4 | 1 | 4 | 0 | 0 | 1 | 0 |
| 50.01491 | 8.88 | -3.1 | 4 | 2 | 0 | 0 | 0 | 4 |
| 51.0228 | 14.79 | -1.7 | 4 | 3 | 0 | 0 | 0 | 3.5 |
| 52.03064 | 4.96 | -1.4 | 4 | 4 | 0 | 0 | 0 | 3 |
| 53.03851 | 13.57 | -0.6 | 4 | 5 | 0 | 0 | 0 | 2.5 |
| 55.01773 | 3.16 | -1.3 | 3 | 3 | 0 | 1 | 0 | 2.5 |
| 55.05427 | 16.86 | 1.5 | 4 | 7 | 0 | 0 | 0 | 1.5 |
| 56.06202 | 3.36 | 0.1 | 4 | 8 | 0 | 0 | 0 | 1 |
| 57.06988 | 4.32 | 0.7 | 4 | 9 | 0 | 0 | 0 | 0.5 |
| 57.98719 | 7.28 | 0.9 | 2 | 2 | 0 | 0 | 1 | 2 |
| 58.99473 | 25.34 | -3.9 | 2 | 3 | 0 | 0 | 1 | 1.5 |
| 61.0101 | 5.19 | -8.4 | 2 | 5 | 0 | 0 | 1 | 0.5 |
| 62.01477 | 4.14 | -4.7 | 5 | 2 | 0 | 0 | 0 | 5 |
| 62.01827 | 7.79 | -2.7 | 2 | 6 | 0 | 0 | 1 | 0 |
| 63.02306 | 8.58 | 2.7 | 5 | 3 | 0 | 0 | 0 | 4.5 |
| 63.96137 | 60.79 | 0.8 | 0 | 0 | 0 | 2 | 1 | 1 |
| 65.03864 | 18.88 | 1.5 | 5 | 5 | 0 | 0 | 0 | 3.5 |
| 66.04621 | 10.34 | -2.3 | 5 | 6 | 0 | 0 | 0 | 3 |
| 67.05441 | 12.30 | 3.3 | 5 | 7 | 0 | 0 | 0 | 2.5 |
| 68.97916 | 7.23 | -2.2 | 3 | 1 | 0 | 0 | 1 | 3.5 |
| 69.0701 | 6.85 | 3.8 | 5 | 9 | 0 | 0 | 0 | 1.5 |
| 70.99459 | 7.17 | -5.2 | 3 | 3 | 0 | 0 | 1 | 2.5 |
| 75.02577 | 3.19 | -6.5 | 3 | 7 | 0 | 0 | 1 | 0.5 |
| 77.03817 | 21.80 | -4.8 | 6 | 5 | 0 | 0 | 0 | 4.5 |
| 78.0464 | 8.17 | 0.5 | 6 | 6 | 0 | 0 | 0 | 4 |
| 79.0542 | 9.51 | 0.1 | 6 | 7 | 0 | 0 | 0 | 3.5 |
| 81.06971 | 6.73 | -1.6 | 6 | 9 | 0 | 0 | 0 | 2.5 |
| 85.01065 | 4.62 | 0.5 | 4 | 5 | 0 | 0 | 1 | 2.5 |
| 91.05451 | 21.43 | 3.5 | 7 | 7 | 0 | 0 | 0 | 4.5 |
| 93.06969 | 3.00 | -1.6 | 7 | 9 | 0 | 0 | 0 | 3.5 |
| 94.04113 | 25.94 | -1.6 | 6 | 6 | 0 | 1 | 0 | 4 |
| 95.08537 | 3.71 | -1.3 | 7 | 11 | 0 | 0 | 0 | 2.5 |
| 97.01017 | 30.50 | -4.5 | 5 | 5 | 0 | 0 | 1 | 3.5 |
| 98.01811 | 3.24 | -3.3 | 5 | 6 | 0 | 0 | 1 | 3 |
| 99.02612 | 3.20 | -1.4 | 5 | 7 | 0 | 0 | 1 | 2.5 |
| 103.05509 | 3.70 | 8.7 | 8 | 7 | 0 | 0 | 0 | 5.5 |
| 105.07003 | 10.40 | 1.8 | 8 | 9 | 0 | 0 | 0 | 4.5 |
| 109.0106 | 3.11 | -0.1 | 6 | 5 | 0 | 0 | 1 | 4.5 |
| 110.01792 | 6.20 | -4.7 | 6 | 6 | 0 | 0 | 1 | 4 |
| 111.02592 | 100.00 | -3.1 | 6 | 7 | 0 | 0 | 1 | 3.5 |
| 112.03167 | 7.44 | 8.5 | 9 | 4 | 0 | 0 | 0 | 8 |
| 112.03281 | 14.01 | -11.4 | 6 | 8 | 0 | 0 | 1 | 3 |
| 113.02286 | 4.53 | -3.8 | 5 | 5 | 0 | 3 | 0 | 3.5 |
| 115.05473 | 8.21 | 4.7 | 9 | 7 | 0 | 0 | 0 | 6.5 |
| 117.06944 | 4.08 | -3.4 | 9 | 9 | 0 | 0 | 0 | 5.5 |
| 119.08527 | 10.00 | -1.8 | 9 | 11 | 0 | 0 | 0 | 4.5 |
| 123.02629 | 11.52 | 0.2 | 7 | 7 | 0 | 0 | 1 | 4.5 |
| 124.03415 | 6.24 | 0.5 | 7 | 8 | 0 | 0 | 1 | 4 |
| 125.04233 | 62.95 | 3.3 | 7 | 9 | 0 | 0 | 1 | 3.5 |
| 126.04513 | 4.13 | -9.8 | 10 | 6 | 0 | 0 | 0 | 8 |
| 126.04953 | 17.52 | -1.6 | 7 | 10 | 0 | 0 | 1 | 3 |
| 127.05436 | 3.32 | 1.3 | 10 | 7 | 0 | 0 | 0 | 7.5 |
| 128.06179 | 5.50 | -1.8 | 10 | 8 | 0 | 0 | 0 | 7 |
| 129.07146 | 3.73 | 12.6 | 10 | 9 | 0 | 0 | 0 | 6.5 |
| 131.08547 | 3.28 | -0.1 | 10 | 11 | 0 | 0 | 0 | 5.5 |
| 133.10133 | 5.08 | 1.4 | 10 | 13 | 0 | 0 | 0 | 4.5 |
| 134.01841 | 3.69 | -0.2 | 8 | 6 | 0 | 0 | 1 | 6 |
| 135.0259 | 3.94 | -2.7 | 8 | 7 | 0 | 0 | 1 | 5.5 |
| 137.04227 | 13.33 | 2.6 | 8 | 9 | 0 | 0 | 1 | 4.5 |
| 138.0494 | 8.23 | -2.4 | 8 | 10 | 0 | 0 | 1 | 4 |
| 139.05772 | 32.40 | 1.1 | 8 | 11 | 0 | 0 | 1 | 3.5 |
| 140.0646 | 10.30 | -5.6 | 8 | 12 | 0 | 0 | 1 | 3 |
| 141.06966 | 4.03 | -1.3 | 11 | 9 | 0 | 0 | 0 | 7.5 |
| 147.02584 | 8.57 | -2.9 | 9 | 7 | 0 | 0 | 1 | 6.5 |
| 148.03435 | 3.64 | 1.8 | 9 | 8 | 0 | 0 | 1 | 6 |
| 149.0413 | 3.58 | -4.1 | 9 | 9 | 0 | 0 | 1 | 5.5 |
| 151.05776 | 13.74 | 1.3 | 9 | 11 | 0 | 0 | 1 | 4.5 |
| 152.06392 | 6.19 | -9.6 | 9 | 12 | 0 | 0 | 1 | 4 |
| 153.07322 | 16.17 | 0.1 | 9 | 13 | 0 | 0 | 1 | 3.5 |
| 154.08004 | 6.25 | -6.5 | 9 | 14 | 0 | 0 | 1 | 3 |
| 161.04098 | 7.93 | -5.8 | 10 | 9 | 0 | 0 | 1 | 6.5 |
| 162.04962 | 3.68 | -0.7 | 10 | 10 | 0 | 0 | 1 | 6 |
| 163.05747 | 3.84 | -0.6 | 10 | 11 | 0 | 0 | 1 | 5.5 |
| 165.07304 | 9.17 | -1.0 | 10 | 13 | 0 | 0 | 1 | 4.5 |
| 166.08074 | 3.76 | -1.8 | 10 | 14 | 0 | 0 | 1 | 4 |
| 167.08834 | 8.50 | -3.1 | 10 | 15 | 0 | 0 | 1 | 3.5 |
| 168.09527 | 3.80 | -8.4 | 10 | 16 | 0 | 0 | 1 | 3 |
| 175.05746 | 5.48 | -0.6 | 11 | 11 | 0 | 0 | 1 | 6.5 |
| 177.0731 | 3.46 | -0.6 | 11 | 13 | 0 | 0 | 1 | 5.5 |
| 179.08794 | 6.60 | -5.1 | 11 | 15 | 0 | 0 | 1 | 4.5 |
| 181.10322 | 5.01 | -7.1 | 11 | 17 | 0 | 0 | 1 | 3.5 |
| 189.0719 | 3.78 | -6.9 | 12 | 13 | 0 | 0 | 1 | 6.5 |
| 193.10362 | 4.32 | -4.6 | 12 | 17 | 0 | 0 | 1 | 4.5 |
| 195.11879 | 3.26 | -7.0 | 12 | 19 | 0 | 0 | 1 | 3.5 |

Table S5 Assigned m/z values of the mass loss event between 300-450 °C in the TGA-MS of SBS (Fig 6 **A** and S1 **B**).

| Assigned Exact Mass  [Da] | Relative Intensity  [%] | Mass  Error  [ppm] | C | H | N | O | S | DBE  value |
| --- | --- | --- | --- | --- | --- | --- | --- | --- |
| 40.03093 | 5.45 | 5.4 | 3 | 4 | 0 | 0 | 0 | 2 |
| 41.03881 | 45.21 | 6.6 | 3 | 5 | 0 | 0 | 0 | 1.5 |
| 42.04647 | 11.65 | 2.5 | 3 | 6 | 0 | 0 | 0 | 1 |
| 43.01807 | 4.54 | 6.2 | 2 | 3 | 0 | 1 | 0 | 1.5 |
| 43.05417 | 20.62 | -0.4 | 3 | 7 | 0 | 0 | 0 | 0.5 |
| 43.98938 | 54.44 | 3.1 | 1 | 0 | 0 | 2 | 0 | 2 |
| 44.97924 | 32.21 | -1.6 | 1 | 1 | 0 | 0 | 1 | 1.5 |
| 47.96655 | 9.66 | 3.1 | 0 | 0 | 0 | 1 | 1 | 1 |
| 50.01483 | 10.28 | -4.7 | 4 | 2 | 0 | 0 | 0 | 4 |
| 51.02302 | 18.09 | 2.6 | 4 | 3 | 0 | 0 | 0 | 3.5 |
| 52.03046 | 5.51 | -4.9 | 4 | 4 | 0 | 0 | 0 | 3 |
| 53.03868 | 18.05 | 2.7 | 4 | 5 | 0 | 0 | 0 | 2.5 |
| 54.04648 | 3.13 | 2.1 | 4 | 6 | 0 | 0 | 0 | 2 |
| 55.05438 | 24.59 | 3.5 | 4 | 7 | 0 | 0 | 0 | 1.5 |
| 56.0623 | 10.29 | 5.1 | 4 | 8 | 0 | 0 | 0 | 1 |
| 56.97942 | 3.10 | 1.9 | 2 | 1 | 0 | 0 | 1 | 2.5 |
| 57.07002 | 8.02 | 3.2 | 4 | 9 | 0 | 0 | 0 | 0.5 |
| 57.98731 | 9.24 | 3.0 | 2 | 2 | 0 | 0 | 1 | 2 |
| 58.99514 | 30.03 | 3.0 | 2 | 3 | 0 | 0 | 1 | 1.5 |
| 59.96655 | 4.07 | 2.5 | 1 | 0 | 0 | 1 | 1 | 2 |
| 62.01543 | 4.71 | 5.9 | 5 | 2 | 0 | 0 | 0 | 5 |
| 63.02275 | 10.88 | -2.2 | 5 | 3 | 0 | 0 | 0 | 4.5 |
| 63.94331 | 10.58 | -3.9 | 0 | 0 | 0 | 0 | 2 | 1 |
| 63.96138 | 19.72 | 1.0 | 0 | 0 | 0 | 2 | 1 | 1 |
| 65.03829 | 15.14 | -3.8 | 5 | 5 | 0 | 0 | 0 | 3.5 |
| 66.04631 | 4.58 | -0.8 | 5 | 6 | 0 | 0 | 0 | 3 |
| 67.05445 | 15.92 | 3.9 | 5 | 7 | 0 | 0 | 0 | 2.5 |
| 68.97939 | 13.70 | 1.1 | 3 | 1 | 0 | 0 | 1 | 3.5 |
| 69.06983 | 9.60 | -0.1 | 5 | 9 | 0 | 0 | 0 | 1.5 |
| 69.98686 | 3.26 | -3.9 | 3 | 2 | 0 | 0 | 1 | 3 |
| 70.07755 | 4.47 | -1.6 | 5 | 10 | 0 | 0 | 0 | 1 |
| 70.99489 | 11.18 | -1.0 | 3 | 3 | 0 | 0 | 1 | 2.5 |
| 74.01478 | 3.73 | -3.8 | 6 | 2 | 0 | 0 | 0 | 6 |
| 75.94345 | 7.75 | -1.4 | 1 | 0 | 0 | 0 | 2 | 2 |
| 77.03866 | 28.64 | 1.6 | 6 | 5 | 0 | 0 | 0 | 4.5 |
| 78.04644 | 10.13 | 1.0 | 6 | 6 | 0 | 0 | 0 | 4 |
| 79.05469 | 13.79 | 6.3 | 6 | 7 | 0 | 0 | 0 | 3.5 |
| 81.06995 | 7.89 | 1.4 | 6 | 9 | 0 | 0 | 0 | 2.5 |
| 83.08526 | 3.16 | -2.8 | 6 | 11 | 0 | 0 | 0 | 1.5 |
| 84.00243 | 3.60 | -4.2 | 4 | 4 | 0 | 0 | 1 | 3 |
| 85.01001 | 6.07 | -7.1 | 4 | 5 | 0 | 0 | 1 | 2.5 |
| 89.03843 | 4.77 | -1.2 | 7 | 5 | 0 | 0 | 0 | 5.5 |
| 91.05381 | 35.26 | -4.2 | 7 | 7 | 0 | 0 | 0 | 4.5 |
| 92.06202 | 4.62 | 0.1 | 7 | 8 | 0 | 0 | 0 | 4 |
| 93.06996 | 4.06 | 1.3 | 7 | 9 | 0 | 0 | 0 | 3.5 |
| 95.08546 | 4.43 | -0.3 | 7 | 11 | 0 | 0 | 0 | 2.5 |
| 97.01019 | 44.78 | -4.3 | 5 | 5 | 0 | 0 | 1 | 3.5 |
| 98.01859 | 5.31 | 1.6 | 5 | 6 | 0 | 0 | 1 | 3 |
| 99.02635 | 3.35 | 0.9 | 5 | 7 | 0 | 0 | 1 | 2.5 |
| 103.05453 | 5.38 | 3.3 | 8 | 7 | 0 | 0 | 0 | 5.5 |
| 105.06968 | 13.86 | -1.5 | 8 | 9 | 0 | 0 | 0 | 4.5 |
| 106.07755 | 3.83 | -1.1 | 8 | 10 | 0 | 0 | 0 | 4 |
| 109.0108 | 5.67 | 1.7 | 6 | 5 | 0 | 0 | 1 | 4.5 |
| 110.01842 | 8.55 | -0.1 | 6 | 6 | 0 | 0 | 1 | 4 |
| 111.02659 | 100.00 | 3.0 | 6 | 7 | 0 | 0 | 1 | 3.5 |
| 112.03024 | 6.51 | -4.2 | 9 | 4 | 0 | 0 | 0 | 8 |
| 112.03419 | 19.48 | 0.9 | 6 | 8 | 0 | 0 | 1 | 3 |
| 113.02225 | 4.48 | -9.2 | 5 | 5 | 0 | 3 | 0 | 3.5 |
| 113.99604 | 3.43 | 11.5 | 4 | 2 | 0 | 4 | 0 | 4 |
| 115.0545 | 12.41 | 2.7 | 9 | 7 | 0 | 0 | 0 | 6.5 |
| 117.07002 | 4.39 | 1.5 | 9 | 9 | 0 | 0 | 0 | 5.5 |
| 119.08555 | 7.36 | 0.5 | 9 | 11 | 0 | 0 | 0 | 4.5 |
| 121.01108 | 5.57 | 3.9 | 7 | 5 | 0 | 0 | 1 | 5.5 |
| 123.02588 | 33.10 | -3.1 | 7 | 7 | 0 | 0 | 1 | 4.5 |
| 124.03398 | 14.18 | -0.9 | 7 | 8 | 0 | 0 | 1 | 4 |
| 125.00601 | 5.19 | 3.9 | 6 | 5 | 0 | 1 | 1 | 4.5 |
| 125.04176 | 54.73 | -1.2 | 7 | 9 | 0 | 0 | 1 | 3.5 |
| 126.04991 | 20.23 | 1.4 | 7 | 10 | 0 | 0 | 1 | 3 |
| 127.05431 | 4.54 | 1.0 | 10 | 7 | 0 | 0 | 0 | 7.5 |
| 128.06298 | 7.97 | 7.5 | 10 | 8 | 0 | 0 | 0 | 7 |
| 129.07057 | 5.24 | 5.7 | 10 | 9 | 0 | 0 | 0 | 6.5 |
| 133.10145 | 3.41 | 2.3 | 10 | 13 | 0 | 0 | 0 | 4.5 |
| 134.01823 | 7.99 | -1.5 | 8 | 6 | 0 | 0 | 1 | 6 |
| 135.0268 | 6.89 | 4.0 | 8 | 7 | 0 | 0 | 1 | 5.5 |
| 137.04143 | 24.49 | -3.5 | 8 | 9 | 0 | 0 | 1 | 4.5 |
| 138.04834 | 13.87 | -10.1 | 8 | 10 | 0 | 0 | 1 | 4 |
| 139.05718 | 24.31 | -2.7 | 8 | 11 | 0 | 0 | 1 | 3.5 |
| 140.06391 | 9.71 | -10.5 | 8 | 12 | 0 | 0 | 1 | 3 |
| 141.07002 | 6.10 | 1.3 | 11 | 9 | 0 | 0 | 0 | 7.5 |
| 147.02608 | 14.59 | -1.2 | 9 | 7 | 0 | 0 | 1 | 6.5 |
| 148.03402 | 6.22 | -0.4 | 9 | 8 | 0 | 0 | 1 | 6 |
| 149.04185 | 5.29 | -0.4 | 9 | 9 | 0 | 0 | 1 | 5.5 |
| 151.0574 | 15.66 | -1.1 | 9 | 11 | 0 | 0 | 1 | 4.5 |
| 152.06592 | 10.41 | 3.5 | 9 | 12 | 0 | 0 | 1 | 4 |
| 152.98282 | 6.69 | 0.9 | 7 | 5 | 0 | 0 | 2 | 5.5 |
| 153.07375 | 12.53 | 3.5 | 9 | 13 | 0 | 0 | 1 | 3.5 |
| 154.08044 | 5.52 | -3.9 | 9 | 14 | 0 | 0 | 1 | 3 |
| 161.04241 | 10.99 | 3.1 | 10 | 9 | 0 | 0 | 1 | 6.5 |
| 162.0498 | 5.59 | 0.4 | 10 | 10 | 0 | 0 | 1 | 6 |
| 163.05691 | 5.11 | -4.0 | 10 | 11 | 0 | 0 | 1 | 5.5 |
| 163.99287 | 3.19 | 1.5 | 8 | 4 | 0 | 2 | 1 | 7 |
| 165.0721 | 11.27 | -6.7 | 10 | 13 | 0 | 0 | 1 | 4.5 |
| 166.0795 | 4.80 | -9.2 | 10 | 14 | 0 | 0 | 1 | 4 |
| 166.99913 | 6.11 | 4.8 | 8 | 7 | 0 | 0 | 2 | 5.5 |
| 167.0879 | 6.13 | -5.8 | 10 | 15 | 0 | 0 | 1 | 3.5 |
| 171.02561 | 3.32 | -3.8 | 11 | 7 | 0 | 0 | 1 | 8.5 |
| 173.04129 | 3.15 | -3.6 | 11 | 9 | 0 | 0 | 1 | 7.5 |
| 175.05712 | 6.38 | -2.5 | 11 | 11 | 0 | 0 | 1 | 6.5 |
| 176.06371 | 4.06 | -9.5 | 11 | 12 | 0 | 0 | 1 | 6 |
| 177.07276 | 4.34 | -2.5 | 11 | 13 | 0 | 0 | 1 | 5.5 |
| 179.08937 | 6.59 | 2.8 | 11 | 15 | 0 | 0 | 1 | 4.5 |
| 181.01381 | 3.00 | -1.0 | 9 | 9 | 0 | 0 | 2 | 5.5 |
| 181.1042 | 3.45 | -1.7 | 11 | 17 | 0 | 0 | 1 | 3.5 |
| 189.07375 | 4.49 | 2.8 | 12 | 13 | 0 | 0 | 1 | 6.5 |
| 191.08918 | 3.53 | 1.7 | 12 | 15 | 0 | 0 | 1 | 5.5 |
| 193.01408 | 3.27 | 0.5 | 10 | 9 | 0 | 0 | 2 | 6.5 |
| 193.10419 | 4.18 | -1.7 | 12 | 17 | 0 | 0 | 1 | 4.5 |
| 207.03048 | 3.11 | 4.1 | 11 | 11 | 0 | 0 | 2 | 6.5 |
| 213.99006 | 3.75 | 2.0 | 11 | 2 | 0 | 5 | 0 | 11 |

Table S6 Assigned m/z values of the mass loss event between 100-250 °C in the TGA-MS of the L/L extract (Fig 6 **C** and S1 **C**).

| Assigned Exact Mass  [Da] | Relative Intensity  [%] | Mass  Error  [ppm] | C | H | N | O | S | DBE  value |
| --- | --- | --- | --- | --- | --- | --- | --- | --- |
| 41.03864 | 27.34 | 2.5 | 3 | 5 | 0 | 0 | 0 | 1.5 |
| 43.05437 | 9.01 | 4.2 | 3 | 7 | 0 | 0 | 0 | 0.5 |
| 43.98931 | 20.22 | 1.5 | 1 | 0 | 0 | 2 | 0 | 2 |
| 44.97946 | 23.45 | 3.3 | 1 | 1 | 0 | 0 | 1 | 1.5 |
| 46.99497 | 4.49 | 0.2 | 1 | 3 | 0 | 0 | 1 | 0.5 |
| 47.96657 | 42.97 | 3.5 | 0 | 0 | 0 | 1 | 1 | 1 |
| 50.01512 | 5.68 | 1.1 | 4 | 2 | 0 | 0 | 0 | 4 |
| 51.02291 | 11.24 | 0.4 | 4 | 3 | 0 | 0 | 0 | 3.5 |
| 52.03077 | 3.34 | 1.1 | 4 | 4 | 0 | 0 | 0 | 3 |
| 53.03867 | 12.94 | 2.5 | 4 | 5 | 0 | 0 | 0 | 2.5 |
| 55.05433 | 15.79 | 2.6 | 4 | 7 | 0 | 0 | 0 | 1.5 |
| 56.0617 | 3.64 | -5.6 | 4 | 8 | 0 | 0 | 0 | 1 |
| 57.07011 | 3.69 | 4.7 | 4 | 9 | 0 | 0 | 0 | 0.5 |
| 57.98742 | 6.00 | 4.9 | 2 | 2 | 0 | 0 | 1 | 2 |
| 58.99495 | 22.81 | -0.2 | 2 | 3 | 0 | 0 | 1 | 1.5 |
| 63.0229 | 5.53 | 0.2 | 5 | 3 | 0 | 0 | 0 | 4.5 |
| 63.96125 | 88.83 | -1.0 | 0 | 0 | 0 | 2 | 1 | 1 |
| 65.03859 | 9.71 | 0.8 | 5 | 5 | 0 | 0 | 0 | 3.5 |
| 67.0541 | 11.50 | -1.3 | 5 | 7 | 0 | 0 | 0 | 2.5 |
| 68.97966 | 7.07 | 5.1 | 3 | 1 | 0 | 0 | 1 | 3.5 |
| 69.07009 | 6.58 | 3.6 | 5 | 9 | 0 | 0 | 0 | 1.5 |
| 70.995 | 7.11 | 0.5 | 3 | 3 | 0 | 0 | 1 | 2.5 |
| 77.03855 | 18.19 | 0.1 | 6 | 5 | 0 | 0 | 0 | 4.5 |
| 78.04606 | 6.22 | -3.9 | 6 | 6 | 0 | 0 | 0 | 4 |
| 79.0542 | 7.65 | 0.1 | 6 | 7 | 0 | 0 | 0 | 3.5 |
| 81.06929 | 5.81 | -6.8 | 6 | 9 | 0 | 0 | 0 | 2.5 |
| 85.00995 | 4.74 | -7.8 | 4 | 5 | 0 | 0 | 1 | 2.5 |
| 91.0542 | 17.24 | 0.1 | 7 | 7 | 0 | 0 | 0 | 4.5 |
| 95.08558 | 3.21 | 1.0 | 7 | 11 | 0 | 0 | 0 | 2.5 |
| 97.01075 | 37.76 | 1.4 | 5 | 5 | 0 | 0 | 1 | 3.5 |
| 98.01844 | 5.08 | 0.0 | 5 | 6 | 0 | 0 | 1 | 3 |
| 99.02653 | 3.02 | 2.7 | 5 | 7 | 0 | 0 | 1 | 2.5 |
| 105.07043 | 6.39 | 5.6 | 8 | 9 | 0 | 0 | 0 | 4.5 |
| 110.01878 | 5.69 | 3.1 | 6 | 6 | 0 | 0 | 1 | 4 |
| 111.02649 | 100.00 | 2.1 | 6 | 7 | 0 | 0 | 1 | 3.5 |
| 112.03026 | 7.53 | -4.1 | 9 | 4 | 0 | 0 | 0 | 8 |
| 112.03433 | 16.38 | 2.2 | 6 | 8 | 0 | 0 | 1 | 3 |
| 113.02229 | 4.53 | -8.8 | 5 | 5 | 0 | 3 | 0 | 3.5 |
| 115.05302 | 6.17 | -10.2 | 9 | 7 | 0 | 0 | 0 | 6.5 |
| 119.08587 | 6.04 | 3.2 | 9 | 11 | 0 | 0 | 0 | 4.5 |
| 123.02571 | 9.64 | -4.5 | 7 | 7 | 0 | 0 | 1 | 4.5 |
| 124.03364 | 5.51 | -3.6 | 7 | 8 | 0 | 0 | 1 | 4 |
| 125.04183 | 64.21 | -0.6 | 7 | 9 | 0 | 0 | 1 | 3.5 |
| 126.04608 | 4.62 | -2.3 | 10 | 6 | 0 | 0 | 0 | 8 |
| 126.04987 | 18.53 | 1.1 | 7 | 10 | 0 | 0 | 1 | 3 |
| 127.05365 | 3.06 | -4.2 | 10 | 7 | 0 | 0 | 0 | 7.5 |
| 128.06273 | 4.14 | 5.6 | 10 | 8 | 0 | 0 | 0 | 7 |
| 133.10073 | 3.57 | -3.1 | 10 | 13 | 0 | 0 | 0 | 4.5 |
| 135.02609 | 3.01 | -1.3 | 8 | 7 | 0 | 0 | 1 | 5.5 |
| 137.04166 | 11.03 | -1.8 | 8 | 9 | 0 | 0 | 1 | 4.5 |
| 138.05 | 6.20 | 1.9 | 8 | 10 | 0 | 0 | 1 | 4 |
| 139.05714 | 30.49 | -3.0 | 8 | 11 | 0 | 0 | 1 | 3.5 |
| 140.06544 | 10.72 | 0.4 | 8 | 12 | 0 | 0 | 1 | 3 |
| 141.06936 | 3.19 | -3.4 | 11 | 9 | 0 | 0 | 0 | 7.5 |
| 147.02612 | 6.75 | -1.0 | 9 | 7 | 0 | 0 | 1 | 6.5 |
| 151.05783 | 10.06 | 1.8 | 9 | 11 | 0 | 0 | 1 | 4.5 |
| 152.06484 | 5.13 | -3.6 | 9 | 12 | 0 | 0 | 1 | 4 |
| 153.07353 | 14.69 | 2.1 | 9 | 13 | 0 | 0 | 1 | 3.5 |
| 154.08065 | 6.23 | -2.5 | 9 | 14 | 0 | 0 | 1 | 3 |
| 161.04207 | 5.74 | 1.0 | 10 | 9 | 0 | 0 | 1 | 6.5 |
| 163.05834 | 3.01 | 4.8 | 10 | 11 | 0 | 0 | 1 | 5.5 |
| 165.07335 | 8.27 | 0.8 | 10 | 13 | 0 | 0 | 1 | 4.5 |
| 167.08817 | 7.65 | -4.1 | 10 | 15 | 0 | 0 | 1 | 3.5 |
| 168.09629 | 3.63 | -2.4 | 10 | 16 | 0 | 0 | 1 | 3 |
| 175.05726 | 4.10 | -1.7 | 11 | 11 | 0 | 0 | 1 | 6.5 |
| 179.08945 | 6.18 | 3.3 | 11 | 15 | 0 | 0 | 1 | 4.5 |
| 181.1038 | 4.53 | -3.9 | 11 | 17 | 0 | 0 | 1 | 3.5 |
| 193.10401 | 4.18 | -2.6 | 12 | 17 | 0 | 0 | 1 | 4.5 |

Table S7 Relative intensity, measured via ESI(-) FT-ICR MS, of compound classes not detected via GC×GC.

| sample | Other  CHO_3_S_1-2_ species [%] | CHO_3_S_3_ [%] | CHO_3_S_4_ [%] | sum of  other  sulfonates [%] |
| --- | --- | --- | --- | --- |
| 1 | 2.7 | 6.9 | 1.1 | 10.6 |
| 2 | 3.3 | 6.8 | 0.8 | 10.8 |
| 3 | 3.9 | 8.1 | 1.4 | 13.5 |
| mean | 3.3 | 7.3 | 1.1 | 11.6 |
| Standard deviation of the mean | 0.6 | 0.7 | 0.3 | 1.6 |

Table S8 Relative intensity of the elemental composition for SBS measured via DI ESI(‑) FT-ICR MS. Average of n=3 measurements in % normalised to the summed ion count.

| sample | CH  O_1_S_1_ | CH  O_1_S_2_ | CH  O_3_S_1_ | CH  O_3_S_2_ | CH  O_3_S_3_ | CH  O_3_S_4_ | CH  O_4_S_1_ | CH  N_1_ | CH  N_1_S_1_ | CH  O_1_ | CH  O_2_ | others |
| --- | --- | --- | --- | --- | --- | --- | --- | --- | --- | --- | --- | --- |
| SBS | 0.27 | 0.01 | 12.2 | 76.6 | 7.27 | 1.09 | 0.39 | 0.00 | 0.00 | 0.04 | 0.59 | 1.58 |
